# Organic amendments as partial replacements for synthetic fertilizers in foxtail millet

**DOI:** 10.1186/s12870-026-08660-1

**Published:** 2026-04-07

**Authors:** Mengen Nie, Lijie Zhao, Genlan Han, Shuqi Dong, Huiling Du, Xiangyang Yuan, Yanjun Yang, Tingting Mu

**Affiliations:** 1 College of Agronomy, Shanxi Agricultural University, Jinzhong, 030800 Shanxi China; 2 Center for Agricultural Genetic Resources Research, Shanxi Agricultural University, Taiyuan, 030000 Shanxi China; 3 College of Agriculture, Yuncheng Vocational and Technical University, Yuncheng, 044000 Shanxi China; 4 Lixian Bureau of Science and Technology, Longnan, 742200 Gansu China; 5 Public Research and Development Center for Featured Coarse Gereals on the Loess Plateau, Ministry of Agriculture and Rural Affairs, Shanxi Agricultural University, No.1, Mingxian South Road, Taigu District, Jinzhong, 030800 Shanxi China; 6 Shanxi Institute of Functional Agriculture, Shanxi Agricultural University, Jinzhong, 030800 Shanxi China; 7 College of Biological Sciences and Technology, Jinzhong University, Jinzhong, 030600 Shanxi China

**Keywords:** Partial organic replacement, Animal manure types, Soil physicochemical properties, Soil microbial community, Nitrogen use efficiency, Foxtail millet

## Abstract

**Background:**

The rapid expansion of the livestock industry has generated large amounts of manure, posing environmental risks. Composted manure improves soil quality (SQI) and crop yield. However, the effects of different manure types on soil ecological multifunctionality (EMF) and microbial functions in foxtail millet fields remain unclear. A two-year experiment with various manure types was conducted to evaluate soil properties, microbial communities, and their effects on nitrogen use efficiency (NUE) in foxtail millet.

**Results:**

At a nitrogen application rate of 225 kg ha^− 1^, the 50% organic replacement treatments (cow manure, pig manure, and sheep manure) mitigated soil alkalization and improved soil nutrient levels and enzyme activities, resulting in higher SQI and EMF, with the pig manure replacement treatment performing best. Improved soil nutrient levels enhanced microbial community diversity and network stability, strengthened soil carbon and nitrogen cycling, and thus improved soil EMF, which directly promoted NUE and yield in foxtail millet. From 2022 to 2023, partial organic substitution treatments increased soil SQI and crop yield by 2.85–4.73 times and 1.03–1.08 times, respectively, compared to the inorganic fertilizer treatment. Soil physicochemical properties and microbial functions (mainly carbon and nitrogen cycling) were key direct drivers of EMF. Notably, key microbial phyla, such as Proteobacteria, were significantly enriched in partial organic replacement treatments, playing a crucial role in stabilizing microbial network structures and maintaining community functions. Additionally, pH changes were the primary driver influencing soil microbial communities.

**Conclusions:**

This study confirmed that partial organic replacement enhanced soil EMF by mitigating soil alkalization, boosting soil nutrient and enzyme levels, and optimizing microbial traits, thereby improving crop NUE and yield. Locally, partial pig manure replacement maximized yield, while partial cow manure replacement optimized grain quality. Our findings provide valuable guidance for scientific fertilization practices and ecological integrity.

**Supplementary Information:**

The online version contains supplementary material available at 10.1186/s12870-026-08660-1.

## Highlights

Organic manure enhances soil microbial diversity and soil ecological multifunctionality (EMF).

Soil elements and microbial function contributed positively to EMF.

Proteobacteria stabilized microbial networks and enhanced C/N cycling functions.

Organic manure application enhanced foxtail millet nitrogen use efficiency by improving soil EMF.

Optimal fertilization employed pig manure for maximum yield and cow manure for better grain quality.

**Supplementary Information:**

The online version contains supplementary material available at 10.1186/s12870-026-08660-1.

## Introduction

The rapid development of animal husbandry has led to increasingly serious environmental pollution caused by poultry and livestock manure [1]. As an environmentally friendly approach, composting can convert animal waste into nutrient-rich organic fertilizers by promoting the activity of microorganisms, thereby providing nutrients for agroecosystems [2]. Many previous studies have demonstrated that partial replacing chemical fertilizers with organic fertilizers (partial organic replacement) can accelerate the recycling of organic waste and minimize the negative environmental impacts of excessive use of chemical fertilizers, making it a viable strategy for advancing sustainable agricultural practices [3–5].

Soil ecological multifunctionality (EMF) refers to the capacity of soil to provide a variety of ecological functions and services [4]. The excessive application of synthetic fertilizers has led to a decline in crop productivity and severe disruption of soil EMF [4–6]. Wu et al. found that, compared with chemical fertilizer alone, organic replacement treatments significantly increased soil nutrient levels, enhanced carbon-acquisition enzyme activities, reduced nitrous oxide emissions, and effectively improved soil quality [7]. A meta-analysis of nearly 40 years of fertilization experiments in China’s upland double-cropping regions demonstrated that the combined application of organic amendments and chemical fertilizers significantly increased crop yield and improved soil quality compared with chemical fertilizers application alone [8]. Abbas et al. suggested that application of organic amendments could significantly increase chlorophyll content and net photosynthetic rate in maize, enhance aboveground nitrogen uptake and biomass accumulation, increase 1000-grain weight, and ultimately improve grain yield [9]. Given the potential of organic replacement to improve soil quality and facilitate nutrient uptake by crops, an increase in soil EMF and crop productivity is generally expected following appropriate organic replacement strategies [3, 4, 6, 7]. Many studies have shown that organic replacement at a ratio of 30%–50% results in the greatest improvement in the yield of various crops like maize, wheat, and rice, while crop yields tend to decline when the organic replacement rate exceeds 60% [3, 4, 7, 8]. Partial organic replacement enhances crop productivity by improving the synchronization of nutrient supply and soil fertility [3]. On the one hand, partial organic replacement can improve soil structure and enhance total and available soil nutrients, thereby reducing or eliminating soil degradation caused by long-term extensive use of chemical fertilizers, and consequently facilitating and promoting improvements in soil EMF and increasing grain yields [4, 7]. Soil salinization in northern China remains a pressing concern, and research has shown that organic acids in manure can neutralize alkaline ions and lower soil pH [6]. Appropriate application of organic fertilizers reduces soil bulk density and increases water retention, thereby fostering favorable soil conditions for the cycling of available chemical nutrients [10]. The release of nutrients from manure in the soil is slow and persistent, and applying organic and inorganic fertilizers together can increase total and available soil nutrients and adjust the balance between soil fertility and nutrient availability, thereby improving soil nutrient utilization efficiency [3, 6]. Soil enzymes fulfill a pivotal function by catalyzing the cycling of soil carbon (C) and nutrients [4]. Manure provides sufficient energy sources for microorganisms and enzymes by injecting large amounts of organic C into the soil [11]. These improvements in soil properties lead to increased EMF, which promotes higher crop productivity and grain yield [4, 12]. On the other hand, partial organic replacement enhances soil EMF and promotes grain yield by altering soil microbial community structure and function [3, 11].

Soil microbes play a vital role in the interaction among soil, plant roots, and the environment, participating in many important ecosystem functions, such as organic material decomposition, nutrient cycles, soil structure formations, as well as plants’ nutrient uptake and growth, consequently acting as key drivers of crop grain yield [4, 6, 12]. Many researchers demonstrated that organic replacement enhances soil EMF by modifying the soil environment and optimizing microbial community structure and function [4, 7, 12]. Hu et al. found that organic fertilizers enriched the relative abundances of bacterial taxa carrying carbon (C) and nitrogen (N) cycling genes, including Proteobacteria and Planctomycetes, while inhibiting the growth of oligotrophic bacteria such as Verrucomicrobia [13]. Li et al. observed that organic fertilizers influenced the functional potential of the soil C and N cycles by shaping keystone taxon activity and the microbial community structure [14]. In addition, organic fertilizers affect interspecific interactions among microorganisms. Liu et al. suggested that the decreased density of soil microbial networks and the reduced positive correlations among genus groups under organic fertilizer treatments may result from diminished resource constraints, and that more negative correlations between microorganisms may be beneficial for resisting pathogen invasion [3]. Research by Shi et al. (2025) showed that organic replacement alleviated the negative impacts of inorganic fertilizer on microbial diversity, stabilized microbial communities, and enriched functional genes involved in C (e.g., *bcrB/C/D*) and N (e.g., *amoB/C*) cycling, thereby enhancing soil EMF [4]. However, previous studies on the application of organic and inorganic fertilizers together had primarily focused on long-term agricultural trial plots [3, 4, 12, 13]. Although some research has demonstrated that the short-term mechanisms of organic fertilizer actions, there is still a lack of research related to the impacts of short-term organic replacement on microbial-mediated rhizosphere processes, as well as the relationships between microbial characteristics, environmental factors, and soil EMF, particularly for characteristic coarse cereals in North China, such as foxtail millet (*Setaria italica* (L.) Beauv.) [6, 11, 15]. Foxtail millet is cultivated across approximately 30 million hectares globally, with an annual production of around 30 million tonnes [16]. China is the world’s second-largest producer, with northern China as the main cultivation region. As a whole-grain raw material with recognized high nutritional value and diverse physiological regulatory functions, foxtail millet is essential to agricultural development in northern China [17]. Therefore, it is crucial to study the short-term effects of partial organic replacement on the soil environment and microbial properties, as well as their impact on soil EMF and crop productivity.

Due to variations in livestock diets, different manure types differ in nutrient levels, antibiotic residues, and heavy metal concentrations. These differences influence soil environmental factors, soil EMF, and microbial community structure and function. So far, no studies have attempted to optimize the types of animal manure compost to increase foxtail millet grain yield. Thus, the aims of our study were to (1) examine the impacts of applying different types of livestock manure on soil quality, microbial community structure and composition, EMF, and foxtail millet yield, compared to chemical fertilizer alone; (2) investigate the effects of different types of livestock manure application on soil microbial functions, as well as the relationships among keystone taxa, environmental factors, and soil C and N functional characteristics; (3) assess the contributions of fertilization regimes, physiochemical properties, and microbial communities to EMF and crop productivity.

Hypotheses: We hypothesize that the combined application of chemical and organic fertilizers will (1) improve soil SQI by increasing nutrient levels and enzyme activities, (2) enhance soil EMF by regulating the structure and function of soil microbial communities, and (3) promote increases in NUE and foxtail millet grain yield through the combined improvement of soil environmental factors and microbial communities, and (4) identify pig manure as the optimal organic replacement for achieving these synergistic effects.

## Materials and methods

### Experimental site

The fertilization experiment began in May 2022 and ended in October 2023 in Xinzhou, Shanxi Province, China (113°27’ E, 39°19’ N; Supplementary Fig. S1A). This region experiences a temperate continental climate, with an annual precipitation of 400 mm and an average temperature of 6.3 °C. The soil type at the location was categorised as Mollisols or Inceptisol based on the USDA soil taxonomy. Prior to sowing in 2021, the soil’s physicochemical parameters were determined as follows: pH (soil: water = 1:2.5) was 8.83, total organic matter was 11.68 g·kg^⁻¹^, total nitrogen (TN) was 0.83 g·kg^⁻¹^, total phosphorus (TP) was 1.30 g·kg^⁻¹^, total potassium (TK) was 11.35 g·kg^⁻¹^, alkaline nitrogen (AN) was 54.36 mg·kg^− 1^, available phosphorus (AP) was 42.87 mg·kg^− 1^, and available potassium (AK) was 67.17 mg·kg^− 1^. The field experiments used the commercial foxtail millet cultivar ‘Zhangza 16’.

### Experimental design

A total of 12 plots (5 m × 6 m) were established using a randomized complete block design, with each plot receiving four treatments and three replicates (the specific experimental settings are shown in Supplementary Fig. S1B). Fertilization trials were conducted in the same field in 2022 and 2023, with corn being the preceding crop. The specific treatments were: (1) NPK (pure inorganic fertilizer); (2) CM (50% inorganic fertilizer N replaced with cow manure N); (3) PM (50% inorganic fertilizer N replaced with pig manure N); (4) SM (50% inorganic fertilizer N replaced with sheep manure N). The field experiment with foxtail millet was conducted with equal N-P₂O₅-K₂O application rates (225, 135, and 112.5 kg ha^− 1^, respectively), with organic N partial replacing chemical N. Inorganic phosphorus (P) and potassium (K) fertilizers were supplemented to meet the target rates when the amounts from organic amendments were insufficient. Synthetic N, P, and K fertilizers were purchased from Yunnan Yuntianhua Co., Ltd., China: urea (46% N), calcium triple superphosphate (44% P₂O₅), and muriate of potash (60% K₂O). Local composted and decayed cow manure (N: P:K = 1.67%:0.43%:0.95% by air-dried weight), pig manure (N: P:K = 2.09%:0.90%:1.12% by air-dried weight), and sheep manure (N: P:K = 2.01%:0.49%:1.32% by air-dried weight) were employed as organic fertilizers. All fertilizers were applied as base fertilizers before planting millet and then incorporated into the topsoil (0–20 cm) using rotary tillage. For a more detailed explanation of the fertilization process, please refer to Supplementary Table 1. Foxtail millet was sown on May 14 and harvested on October 14 in both 2022 and 2023. The rows were spaced 50 cm apart, the holes were spaced 20 cm apart (with two plants per hole), and the seedling density was 400,000 plants ha^− 1^. Local agricultural management practices were employed. Manual weeding was conducted at 30 and 60 days after sowing. Irrigation was applied at the jointing and grain-filling stages of foxtail millet using a high-density drip irrigation system. No plant protection measures were adopted in any treatment.

Samples of rhizosphere soil (top 0–20 cm) were collected at the flowering stage (August 12, BBCH 65) of foxtail millet in 2022 and 2023. Plants were carefully excavated by hand with a spade. Five representative plants per plot were selected for rhizosphere soil analysis. Rhizosphere soil was obtained by gently shaking the roots to dislodge loosely adhering soil particles, followed by brushing off tightly adhering soil from the root surfaces with a sterile brush. Collected soil aliquots were homogenized to form composite samples [18]. Each sample was passed through a 2 mm sterile sieve and split into two subsamples. One subsample was stored at−80 °C for later DNA extraction and analysis (exclusively for samples collected in 2023). The other was air-dried at room temperature for the determination of soil physicochemical properties and extracellular enzyme activities.

Foxtail millet plants were harvested from 3 m² plots on October 14, 2022, and October 14, 2023, respectively, for the determination of grain yield (13% water content). The harvested grains were oven-dried at 65 °C to a constant weight and subsequently pulverized into a fine powder for nutrient content analysis.

### Determination of plant and soil samples

#### Grain quality

Total flavonoid content was measured according to Ma [19].

#### Soil properties

Soil pH was determined at a soil-to-water ratio of 1:2.5 (w/v) using a composite electronic pH probe (PHS-3 C, Leici, Shanghai, China). Total organic carbon (TC) was evaluated via the potassium dichromate (K_2_Cr_2_O_7_) oxidation method. Soil total porosity (SP) was measured according to Bao [20]. Total nitrogen (TN), total phosphorus (TP), and total potassium (TK) were quantified via the Kjeldahl method (KDN−102 C, Shanghai, China), molybdenum blue colorimetry method (U−2900, Hitachi, Japan), and sulfuric-perchloric acid (H₂SO₄-HClO₄) digestion method (FP6400A, Shanghai, China), respectively. Soil ammonium nitrogen (NH₄⁺), nitrate nitrogen (NO₃⁻), available phosphorus (AP), and available potassium (AK) were quantified using four dedicated protocols: indophenol blue colorimetry for NH₄⁺, ultraviolet spectrophotometry for NO₃⁻, the Olsen method for AP, and ammonium acetate extraction for AK. The above indices were measured according to the method of Bao [20]. In this study, an analysis of six soil enzymes associated with C, N, P, and oxidation-reduction cycles was conducted. The activity of soil alkaline phosphatase (ALP) and β-N-acetylglucosaminidase (NAG) was measured using a kit method (BC0280, BC4005; Beijing Solaibao Technology Co., Ltd., Beijing, China). The determination of catalase (CAT), glutaminase (GLS), urease (URE), and invertase (INV) was performed using the potassium permanganate titration method, Nessler’s reagent spectrophotometry method, indophenol-blue colorimetry method, and 3,5-dinitrosalicylic acid method, respectively [21]. Ecological multifunctionality (EMF) was calculated from soil enzyme activities using previously described methods [22, 23].

#### DNA extraction and metagenomic sequencing

The procedures with our previous research [6]. The original data are available in NCBI’s database under the accession PRJNA1373209. Bacterial, fungal, and archaeal diversity indices (observed species index, Shannon index, and Chao1 index) were calculated using QIIME 2.

### Evaluation of soil quality index and N use efficiency

The soil quality index (SQI) was constructed using the total dataset method (10 soil parameters, namely pH, SP, TC, TN, TP, TK, NH_4_^+^, NO_3_^−^, AP, AK) as described by Nabiollahi et al. [24]. Each soil metric was standardized to a value ranging from 0 to 1. The formula was as follows:

1 $$S_{Li}=\frac{x-L}{H-L}$$

Where *x* represents the actual measured value of the soil indicator, H and L represent the highest and lowest values, respectively. *S*_*Li*_ is the linear score of index *i*, ranging from 0 to 1. The SQI score is calculated according to Kuzyakov et al. [25]:

2 $$SQI=0.5\times{{\sum}^{n}_{1}}\times{S}_{Li}{^2}\sin\frac{2{\pi}}{n}$$

Where *n* represents the number of soil properties, and π is 3.14.

N use efficiency (NUE) was calculated according to Cheng et al. [26] as follows

3 $$PFP_N=\frac{Y_{T}}{F_{N}}$$

4 $$AE_{N}=\frac{Y_{T}-Y_{0}}{F_{N}}$$

Where *PFP*_*N*_ and *AE*_*N*_ represent N partial factor productivity and N agronomic efficiency, respectively. *Y*_*T*_ is the foxtail millet grain yield (kg ha^− 1^), *F*_*N*_ represents N application rate (kg ha^− 1^), and *Y*_*0*_ is the foxtail millet grain yield (kg ha^− 1^) in the unfertilized plot.

### Statistical analysis

The normality and homogeneity of variance of the dataset were checked using the Shapiro-Wilk test and Histograms test. One-way ANOVA was performed on data with SPSS 24.0 (IBM Corp., New York, USA), and Duncan’s multiple range test (*p* < 0.05) was applied to compare the four fertilization treatments. Orthogonal contrasts were conducted to compare partial organic replacement treatments with the inorganic NPK control. The bar charts were created using OriginPro 2021 software (IBM Corporation, New York, USA). The linear regression tool (https://www.omicstudio.cn/tool) was used to fit linear models using data from the NPK, CM, PM, and SM treatments. Chiplot software (https://www.chiplot.online) was used to create the correlation analysis heat maps. PCoA (Python 3) was used to characterize differences in microbial community structure across treatments, and PERMANOVA verified the significance of these effects. Venn diagrams and species-level co-occurrence networks [bacteria/fungi: > 0.5% relative abundance; archaea: > 0.01% relative abundance] were generated with OmicStudio (https://www.omicstudio.cn/tool); network relationships were visualized via Gephi (https://gephi.org). Zi-Pi plots were created using Wekemo Bioincloud (https://www.bioincloud.tech). R software was used to compare differentially expressed genes (DEGs) with the CAZy, KEGG, and Gene Ontology databases to annotate their functions. Spsspro software (https://www.spsspro.com) was used to perform a random forest analysis. PLS-PM was built with SPSSAU (https://www.spssau.com), and variance partitioning analysis (VPA) was conducted using OmicStudio (https://www.omicstudio.cn/tool).

## Results

### Soil physicochemical properties

Partial replacement of chemical fertilizers with various types of animal manure significantly affected soil environmental factors (Table 1). Compared with the conventional chemical fertilizer treatment (NPK), partial organic replacement treatments reduced soil pH by 0.05–0.26 units and increased SP by 3.00%–7.58% over the recent two years (2022–2023, *p* < 0.05). Organic replacement treatments significantly enhanced both total and available nutrients. The SM treatment resulted in the largest increases in TC and TP contents: 1.11-fold and 1.26-fold higher in 2022, and 1.14-fold and 1.34-fold higher in 2023, relative to the NPK treatment (*p* < 0.05). Compared to the NPK treatment, the TN, NH_4_^+^, NO_3_^−^, and AP contents were highest in the PM treatment and were 1.15–1.17-fold, 1.13–1.15-fold, 1.22–1.27-fold, and 1.55–1.58-fold higher, respectively (*p* < 0.05). The highest levels of TK and AK were observed in CM treatment, which were 1.10–1.11-fold and 1.28–1.40-fold higher, respectively, than those in NPK treatment. In addition, all organic fertilizer regimes increased soil oxidoreductase (CAT) and hydrolase (INV, NAG, URE, ALP, and GLS) activities compared to the NPK regime. Partial cow manure replacement resulted in the highest CAT and ALP activities, partial pig manure replacement resulted in the highest INV, URE, and GLS activities, and sheep manure replacement resulted in the highest NAG activity. Over the past two years (2022–2023), CM, PM and SM all enhanced soil SQI and EMF compared to NPK, a phenomenon that may be attributable to the incorporation of organic fertilizers (*p* < 0.05). Orthogonal contrasts further showed that partial organic replacement treatments significantly increased soil pH, SP, TC, TN, TP, TK, NH_4_^+^, NO_3_^−^, AP, AK, NAG, INV, CAT, URE, ALP, GLS and SQI compared to the NPK treatment (*p* < 0.01). Subsequent linear regression analyses revealed significantly positive correlations between EMF and SQI across treatments (*p* < 0.01; Supplementary Fig. S2). Specifically, the SQI under CM, PM, and SM increased 2.82–3.94-fold, 4.33–4.65-fold, and 4.41–4.49-fold higher, respectively, compared to NPK from 2022 to 2023 (*p* < 0.05). Among the different organic replacement treatments, EMF was highest in PM, followed by CM and SM, with NPK being the lowest in both years. Additionally, SQI and EMF increased significantly with rising levels of SP, TC, TN, TP, NH_₄_^⁺^, AP, NAG, INV, and URE (*p* < 0.05; Supplementary Fig. S3).

**Table 1:** Effects of partial organic replacement on soil physicochemical properties of foxtail millet in 2022–2023

Soil properties	2022				2023			
	NPK	CM	PM	SM	NPK	CM	PM	SM
pH	8.72± 0.01a	8.60± 0.02c	8.64± 0.02bc	8.67± 0.03b	8.29± 0.02a	8.03± 0.01c	8.14± 0.02b	8.16± 0.04b
Soil total porosity (SP, %)	48.20± 0.43a	49.65± 0.92b	50.28± 0.36b	49.91± 0.70b	47.71± 0.26a	49.95± 0.43b	51.33± 0.36b	50.35± 0.76b
Total organic carbon (TC, g·kg^⁻¹^)	9.62± 0.17c	10.20± 0.17b	10.43± 0.06ab	10.71± 0.18a	9.55± 0.10c	10.22± 0.34b	10.53± 0.24ab	10.90± 0.17a
Total nitrogen (TN, g·kg^⁻¹^)	1.60± 0.01b	1.79± 0.11a	1.83± 0.11a	1.81± 0.08a	1.63± 0.05b	1.83± 0.02a	1.90± 0.02a	1.87± 0.15a
Total phosphorus (TP, g·kg^⁻¹^)	1.49± 0.01d	1.62± 0.03c	1.79± 0.03b	1.88± 0.01a	1.53± 0.03c	1.74± 0.04b	1.90± 0.07ab	2.04± 0.14a
Total potassium (TK, g·kg^⁻¹^)	12.17± 0.35c	13.36± 0.45a	12.64± 0.15bc	12.70± 0.33b	12.37± 0.48b	13.63± 0.22a	13.28± 0.60a	13.33± 0.12a
Ammonium nitrogen (NH_4_^+^, mg·kg^⁻¹^)	17.39± 0.53c	18.29± 0.19bc	19.66± 0.67a	18.96± 0.65ab	17.69± 0.17c	18.76± 0.14bc	20.33± 0.54a	19.47± 1.18ab
Nitrate nitrogen (NO_3_^−^, mg·kg⁻¹)	8.95± 0.10c	9.89± 0.27b	10.92± 0.37a	10.35± 0.70ab	10.15± 0.29c	11.37± 0.12b	12.87± 0.27a	11.93± 0.96b
Available phosphorus (AP, mg·kg^⁻¹^)	31.12± 4.10c	38.66± 1.80b	48.29± 3.29a	40.89± 1.35b	35.88± 4.57c	44.91± 1.96b	56.77± 3.02a	47.98± 2.50b
Available potassium (AK, mg·kg^⁻¹^)	102.17± 1.8c	142.59± 3.52a	95.65± 7.5c	129.7± 3.01b	101.16± 7.41b	129.80± 2.70a	87.41± 1.16c	121.56± 5.73a
Catalase (CAT, U·g^⁻¹^)	0.84± 0.02c	1.03± 0.04a	0.92± 0.04b	1.03± 0.06a	0.91± 0.02d	1.25± 0.02a	1.03± 0.06c	1.16± 0.05b
Invertase (INV, U·g^⁻¹^)	17.87± 0.61b	19.59± 0.79ab	23.38± 2.38a	22.21± 2.28a	18.26± 1.39d	20.06± 0.14c	25.12± 1.31a	23.05± 1.03b
β-N-acetylglucosaminidase (NAG, U·g^⁻¹^)	6.02± 0.14c	6.69± 0.32b	7.03± 0.13ab	7.42± 0.32a	6.46± 0.37c	7.42± 0.19b	7.82± 0.35ab	8.29± 0.19a
Urease (URE, U·g^⁻¹^)	0.39± 0.01c	0.54± 0.06b	0.63± 0.02a	0.50± 0.01b	0.48± 0.02c	0.69± 0.07b	0.90± 0.07a	0.62± 0.01b
Alkaline phosphatase (ALP, U·g^⁻¹^)	0.09± 0.01c	0.15± 0.01a	0.12± 0.02ab	0.11± 0.01bc	0.09± 0.01d	0.16± 0.01a	0.14± 0.01b	0.12± 0.01c
Glutaminase (GLS, U·g^⁻¹^)	25.47± 3.53c	31.80± 1.64b	37.64± 3.82a	28.44± 0.72bc	28.16± 0.82c	35.58± 4.67b	43.33± 3.03a	31.89± 1.55bc
Soil quality index (SQI)	0.36± 0.08b	1.44± 0.3a	1.58± 0.04a	1.64± 0.03a	0.33± 0.07c	0.94± 0.06b	1.56± 0.17a	1.47± 0.31a
Ecological multifunctionality (EMF)	-1.2± 0.15b	0.32± 0.13a	0.65± 0.27a	0.23± 0.38a	-1.23± 0.04c	0.31± 0.04b	0.75± 0.07a	0.17± 0.14b

Data are means ± standard errors (*n* = 3). Different lowercase letters indicate significant differences among treatments (*p* < 0.05, one-way ANOVA with Duncan’s multiple range test). Orthogonal contrasts were performed to compare partial organic replacement treatments with the NPK (pure chemical fertilizer) treatment

*NPK* pure chemical fertilizer, *CM* cow manure replacement, *PM* pig manure replacement, *SM* sheep manure replacement

### N use efficiency, grain yield, and total flavonoid content of foxtail millet

**Fig. 1:**
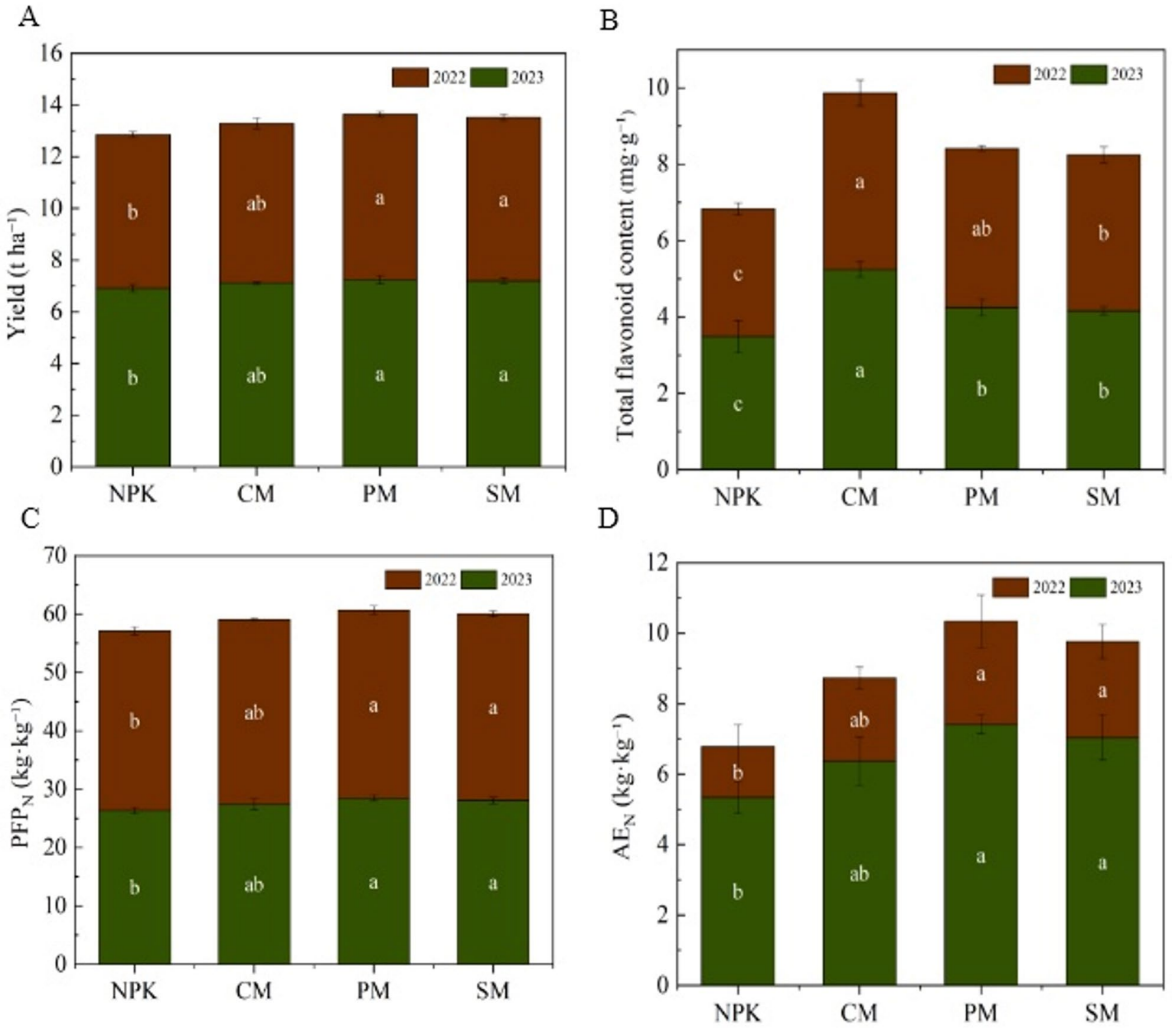
Effects of partial organic replacement on millet yield, quality and N use efficiency. **A** foxtail millet yield, (**B**) total flavonoid content, (**C**) N partial factor productivity (PFP_N_), **D** N agronomic efficiency (AE_N_). Values are means ± standard errors (*n* = 3); bars with different lowercase letters indicate significant differences among treatments at *p* < 0.05 based on one-way ANOVA followed by Duncan’s multiple range test. Orthogonal contrasts were performed to compare partial organic replacement treatments with the NPK treatment (pure chemical fertilizer). NPK: pure chemical fertilizer; CM: cow manure replacement; PM: pig manure replacement; SM: sheep manure replacement

Different fertilization treatments significantly affected crop yield (*p* < 0.05; Fig. 1A). Foxtail millet yield showed a trend of PM > SM > CM > NPK over both years (2022–2023). PM treatment yield was significantly higher than NPK treatment (*p* < 0.05), increasing by 4.81% (2022) and 7.87% (2023). Partial organic replacement also significantly affected grain quality (*p* < 0.05). Partial organic replacement treatments significantly increased the total flavonoid content of foxtail millet grain in both years compared to NPK (*p* < 0.05; Fig. 1B). Total flavonoid content was 1.22–1.38-fold higher in 2022 and 1.20–1.51-fold higher in 2023, with the highest value observed in the CM treatment. Moreover, N use efficiency (NUE) was highly influenced by different fertilization regimes (Fig. 1C and D). Both PFP_N_ and AE_N_ showed a PM > SM > CM trend over the past two years, and were 1.03–1.08-fold and 1.19–2.02-fold higher, respectively, than the NPK treatment. Moreover, linear regression analysis showed that foxtail millet grain yield was significantly positively correlated with SQI, PFP_N_, and AE_N_ (*p* < 0.05; Supplementary Fig. S2C–H). Orthogonal contrasts further showed that partial organic replacement treatments significantly increased foxtail millet grain yield, total flavonoid content, PFP_N_, and AE_N_ compared to the NPK treatment (*p* < 0.01). Spearman’s correlation analyses were used to further clarify the relationship among crop NUE, yield, grain quality, and soil properties (Supplementary Fig. S3). The results revealed the following positive correlations: NUE with TC, NH₄⁺, NO₃⁻, AP, NAG, INV, URE, and SQI; crop yield with SP, TP, NH₄⁺, NO₃⁻, AP, INV, URE, and EMF; and grain quality with pH, TN, CAT, and ALP.

### Microbial diversity and composition

**Fig. 2:**
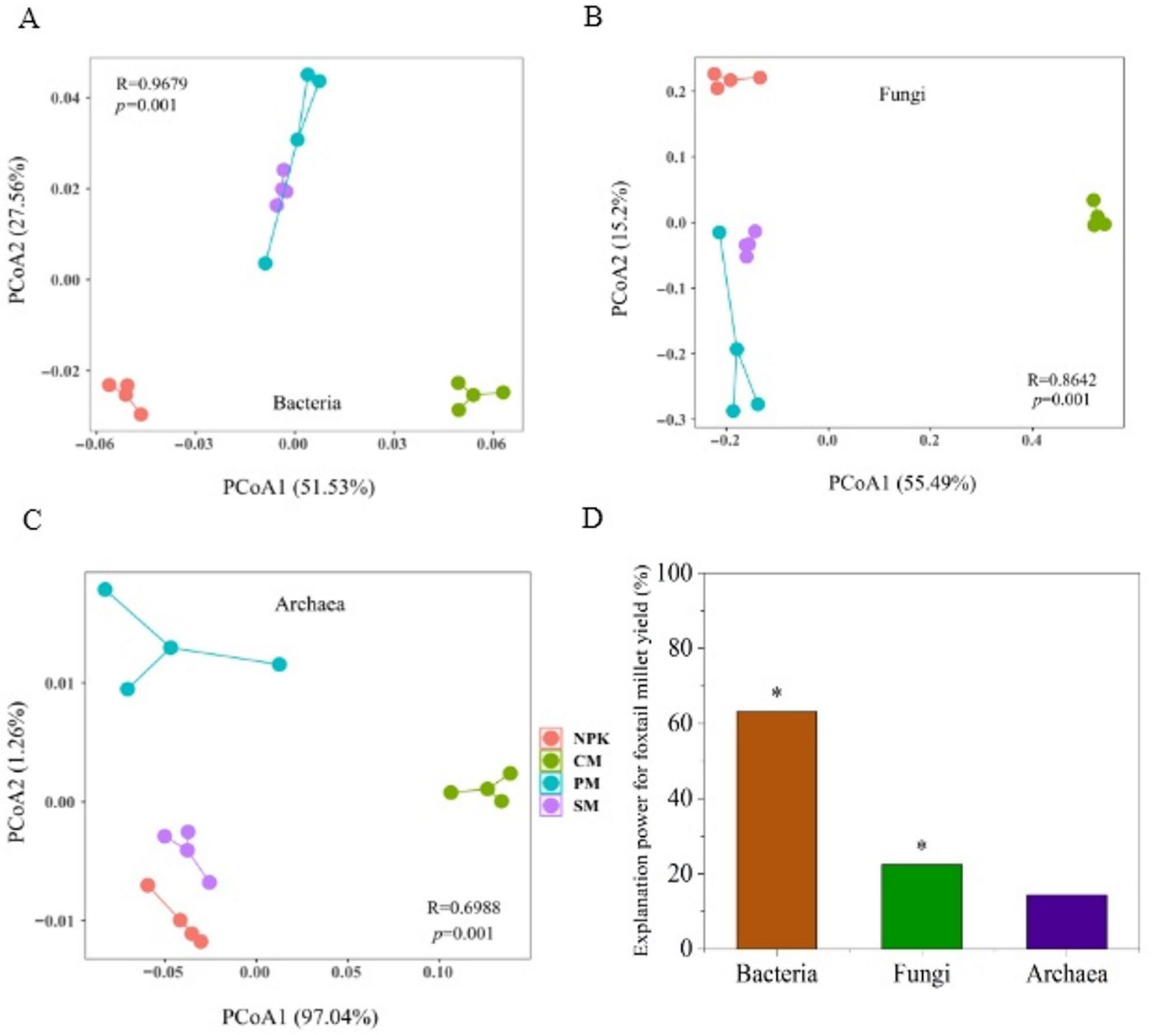
Effects of partial organic replacement on soil microbial diversity and composition. Principal coordinate analysis (PCoA) of bacterial (**A**), fungal (**B**) and archaeal (**C**) communities based on Bray-Curtis distance. Explanatory power of key microbial parameters for grain yield (**D**)

Organic replacement significantly altered soil microbial diversity and community structure in foxtail millet fields (Fig. 2, Supplementary Table 2). Partial organic replacement treatments resulted in higher values of the observed species, Shannon index, and Chao1 index for bacteria, fungi, and archaea compared to the pure inorganic fertilizer treatment (Supplementary Table 2). The PM treatment exhibited the highest values for bacterial observed species, bacterial Chao1 index, and fungal Shannon index. In contrast, the CM treatment showed the highest values for archaeal observed species and archaeal Shannon index. Orthogonal contrasts further revealed that partial organic replacement treatments significantly enhanced soil microbial diversity relative to the NPK treatment (*p* < 0.05). PCoA demonstrated a significant separation (PERMANOVA, *p* = 0.002; Supplementary Table 3) in soil microbial composition between the pure chemical fertilizer (NPK) and the partial organic replacement (CM, PM, SM) treatments, suggesting organic matter significantly affected soil microbial communities in the foxtail millet fields (Fig. 2A–C). CM treatment was clustered on the right side, distinctly separated from the PM and SM treatments, indicating substantial differences in microbial communities between the cow manure regime and both the pig and sheep manure regimes. Conversely, no statistically significant variations were observed in bacterial or fungal communities between the PM and SM regimes. Profiling of the comparative importance of microbial richness, encompassing bacteria, fungi, and archaea, showed that bacterial and fungal richness exerted a substantial influence on grain yield. Of these, the explanatory contribution of bacteria and fungi was 63.2% and 22.5%, respectively (*p* < 0.05; Fig. 2D).

After quality control, 1,580,317, 1,603,604, 1,624,175, and 1,621,858 unigenes were detected by NPK, CM, PM, and SM treatments, respectively (Supplementary Fig. S4A). Soil dominant bacteria (fungi; archaea; the relative abundance > 1%) were mainly composed of Proteobacteria, Acidobacteria, Actinobacteria, Bacteroidetes, Gemmatimonadetes, Chloroflexi, Verrucomicrobia, Candidatus_Rokubacteria, Nitrospirae, and Planctomycetes (Ascomycota, Mucoromycota, Basidiomycota, Chytridiomycota, Zoopagomycota, Microsporidia, and Blastocladiomycota; Thaumarchaeota, Crenarchaeota, Euryarchaeota and Candidatus_Bathyarchaeota), accounting for more than 80% of the relative abundance of the identified phyla (Supplementary Fig. S4, Supplementary Table 4). Compared to NPK, organic replacement treatments decreased the relative abundances of Acidobacteria, Chloroflexi, Candidatus_Rokubacteria, Nitrospirae, Basidiomycota, and Thaumarchaeota, while increasing the relative abundances of Gemmatimonadetes, Chytridiomycota, Microsporidia, Crenarchaeota, Euryarchaeota, and Candidatus_Bathyarchaeota. In addition, compared to NPK, Proteobacteria abundance was markedly higher under CM, while Bacteroidetes and Ascomycota were notably higher under PM (*p* < 0.05). We further analyzed the biomarkers that exhibited a significant difference comparing the pure chemical fertilizer treatment (NPK) and the partial organic replacement treatments (CM, PM, and SM; Supplementary Table 5). A total of 74 shared biomarkers were identified. Of these, 51 biomarkers were notably enriched in the NPK treatment, most of which were classified under the phylum Basidiomycota; and 23 biomarkers were notably enriched in partial organic replacement treatments, mainly belonging to the phylum Chytridiomycota, followed by Proteobacteria, Ascomycota, Microsporidia, and Euryarchaeota (*p* < 0.05).

### Microbial community structure and function

**Fig. 3:**
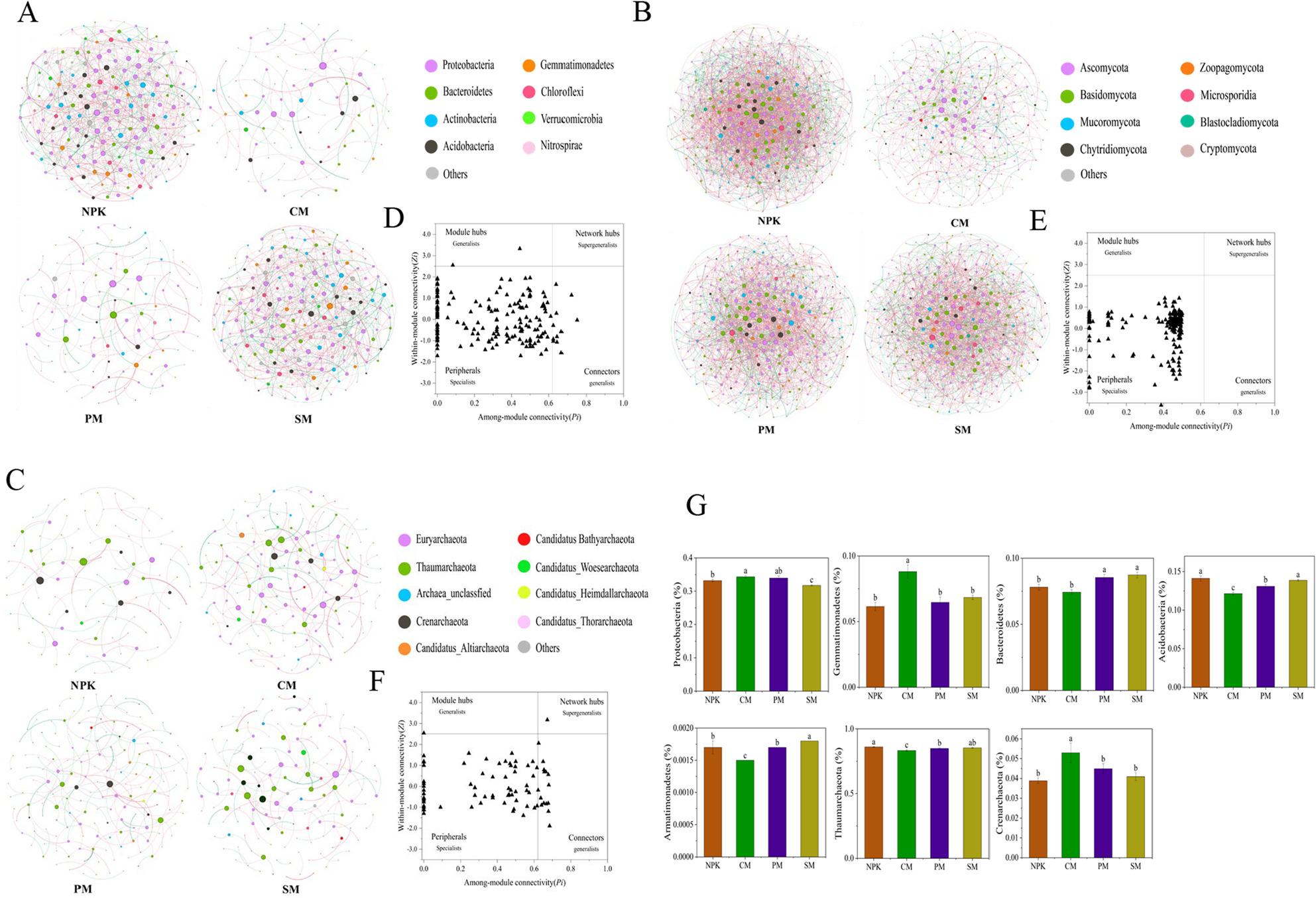
Impacts of NPK, CM, PM and SM on soil microbial co-occurrence networks. Bacterial (**A**), fungal (**B**) and archaeal (**C**) co-occurrence networks at the species level were analyzed, with their topological topological properties shown in Table S6; keystones in the bacterial (**D**), fungal (**E**) and archaeal (**F**) co-occurrence networks; the relative abundance of key phyla corresponding to keystones in the microbial co-occurrence networks (**G**). Values are the means ± standard errors (*n* = 3); bars with different lowercase letters indicate significant differences among treatments at *p* < 0.05 based on one-way ANOVA followed by Duncan’s multiple range test. NPK: pure chemical fertilizer; CM: cow manure replacement; PM: pig manure replacement; SM: sheep manure replacement

Applying pure chemical fertilizer (NPK), cow manure replacement (CM), pig manure replacement (PM), and sheep manure replacement (SM) exhibited different patterns of co-occurrence networks and topological properties (Fig. 3, Supplementary Table 6). Specifically, compared to the NPK treatment, the CM, PM, and SM treatments decreased the number of nodes and edges, network density, and the average degree of the bacterial and fungal co-occurrence networks, while increasing their average path length and modularity (Fig. 3A–C, Supplementary Table 6). In contrast, the archaeal co-occurrence network revealed the opposite. Following network analysis, key nodes composed of bacteria and archaea were identified using ZIPI analysis (Fig. 3D–F, Supplementary Table 7). Notably, the identified keystones belong to seven phyla: Proteobacteria, Gemmatimonadetes, Bacteroidetes, Acidobacteria, Armatimonadetes, Thaumarchaeota (archaea), and Crenarchaeota (archaea; Fig. 3G). The key phyla Acidobacteria, Armatimonadetes, and Thaumarchaeota were most abundant in the NPK and SM treatments, followed by the PM treatment, and had the lowest abundance in the CM treatment. In contrast, the key phyla Proteobacteria, Gemmatimonadetes, and Crenarchaeota showed the highest relative abundance in the CM treatment and the lowest in the NPK treatment. Additionally, the PM and SM treatments significantly elevated Bacteroidetes abundance compared to the NPK treatment (*p* < 0.05). According to Spearman’s correlation analysis (Supplementary Fig. S5), there were significant positive correlations between the key phylum Bacteroidetes and SP, TC, TN, TP, NH₄⁺, NO₃⁻, AP, NAG, INV, SQI, and crop yield (*p* < 0.05). Meanwhile, Proteobacteria and Crenarchaeota exhibited significant positive correlations with URE, ALP, GLS, and EMF (*p* < 0.05). Conversely, Acidobacteria, Armatimonadetes, and Thaumarchaeota displayed negative correlations with ALP and yield.

**Fig. 4:**
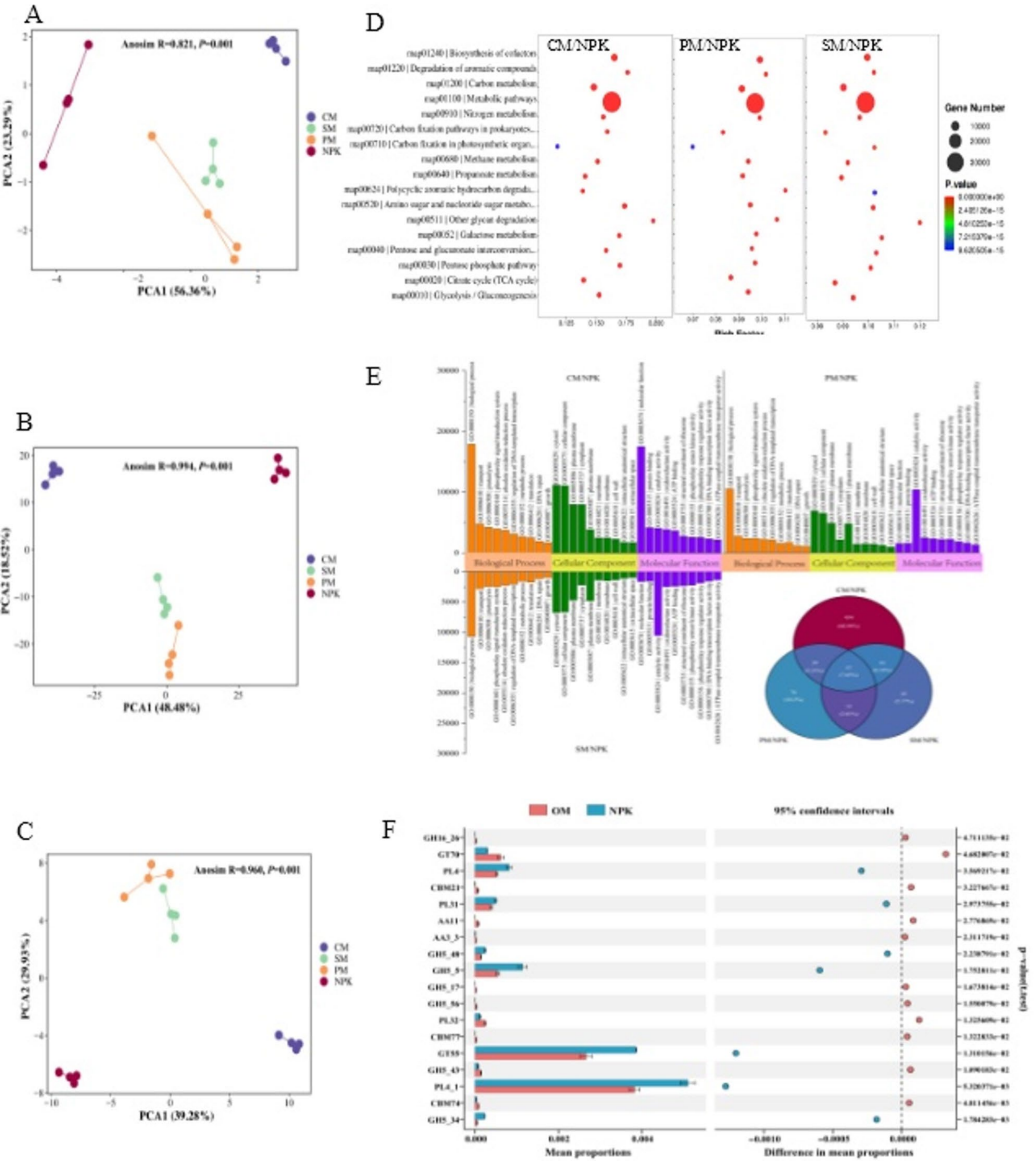
Effects of partial organic replacement on rhizosphere microbial functions. **A**, **B**, and **C** represent the principal component analysis (PCA) of KEGG pathways at level 3, Gene Ontology (GO) enrichment analysis, and carbohydrate active enzymes (CAZymes), respectively. KEGG enrichment analysis (**D**) and GO enrichment analysis (**E**) were performed for differentially expressed genes (DEGs) between the CM, PM, SM, and NPK treatments. **F** The differences in CAZymes between organic and inorganic fertilizer treatments at level 2. NPK: pure chemical fertilizer; CM: cow manure replacement; PM: pig manure replacement; SM: sheep manure replacement; OM: all organic replacement (CM, PM, and SM)

Differential expression genes (DEGs; Supplementary Fig. S6) were identified in the CM/NPK, PM/NPK, and SM/NPK treatments, with 300,456, 169,444, and 178,688 DEGs, respectively (*p* < 0.05). Of these, 154,419, 92,734, and 105,838 were increased, while 146,037, 76,710, and 72,850 were decreased. PCA demonstrated significant differences in the distribution of KEGG (Fig. 4A), GO (Fig. 4B), and CAZymes (Fig. 4C) functions between chemical fertilizer treatment and partial organic replacement treatments (Anosim, *R* ≥ 0.821). KEGG enrichment analysis revealed that, compared to NPK treatment, the partial organic replacement treatments (CM, PM, and SM) significantly reduced the pathways of C fixation in photosynthetic organisms and C fixation pathways in prokaryotes, while significantly increasing the pathways of starch and sucrose metabolism, glycolysis/gluconeogenesis, citrate cycle (TCA cycle), pentose phosphate pathway, pentose and glucuronate interconversions, galactose metabolism, other glycan degradation, amino sugar and nucleotide sugar metabolism, and propanoate metabolism (Fig. 4D, Supplementary Table 8). Meanwhile, the metabolic pathways, including N metabolism, metabolic pathways, and biosynthesis of secondary metabolites, also showed increases in abundance. These changes suggested that applying organic fertilizer enhanced C-consuming activity and N metabolism function. Additionally, CM exhibited the highest number of unigenes increased in response to C consumption, followed by SM and then PM.

GO enrichment analysis of DEGs between chemical fertilizer treatment and partial organic fertilizer replacement treatments identified 611, 187, and 176 GO terms assigned to the three core categories: cellular component (CC), biological process (BP), and molecular function (MF), for the CM vs. NPK, PM vs. NPK, and SM vs. NPK comparisons, respectively (Supplementary Table 9, Fig. 4E). Of these, 57 enriched items were shared in all comparisons and classified into three core categories, with biological process (BP) having the most, followed by molecular function (MF) and cellular component (CC; Supplementary Table 10). Six enriched terms in BP, including “glycophagy”, “positive regulation of glycogen catabolic process”, “photosystem II assembly”, “N-acetylglucosamine biosynthetic process”, “positive regulation of RNA splicing”, and “negative regulation of glucokinase activity”, were significantly increased across all partial organic replacement treatments (Supplementary Table 9, Supplementary Table 10). In addition, 456, 76, and 43 GO terms were enriched in the comparisons of CM, PM, and SM versus NPK, respectively.

Differential analysis of CAZymes revealed that 27, 21, and 10 level-2 CAZymes differed significantly in CM/NPK, PM/NPK, and SM/NPK comparisons, respectively (*p* < 0.05; Supplementary Table 11). Among them, GH5_17 (mannanase) was significantly increased under all partial organic replacement treatments (*p* < 0.05; Fig. 4F), whereas GH5_34 (arabinoxylan) and PL16 (hyaluronate lyase) were more abundant in the NPK treatment. CM treatment showed the highest abundance of GH5_17 and GH16_26, while PM treatment was enriched in GH43_31 and GH5_56. In contrast, NPK treatment had higher abundances of GH5_34, GH5_5, GH43_14, and GT55 (*p* < 0.05; Supplementary Table 12). These results indicated that organic fertilization significantly enhanced carbohydrate-active enzyme activities, especially in the glycoside hydrolase family, with CM treatment showing the strongest response.

### Correlations between soil environmental factors and microbial dominant phyla, biomarkers, and keystones

**Fig. 5:**
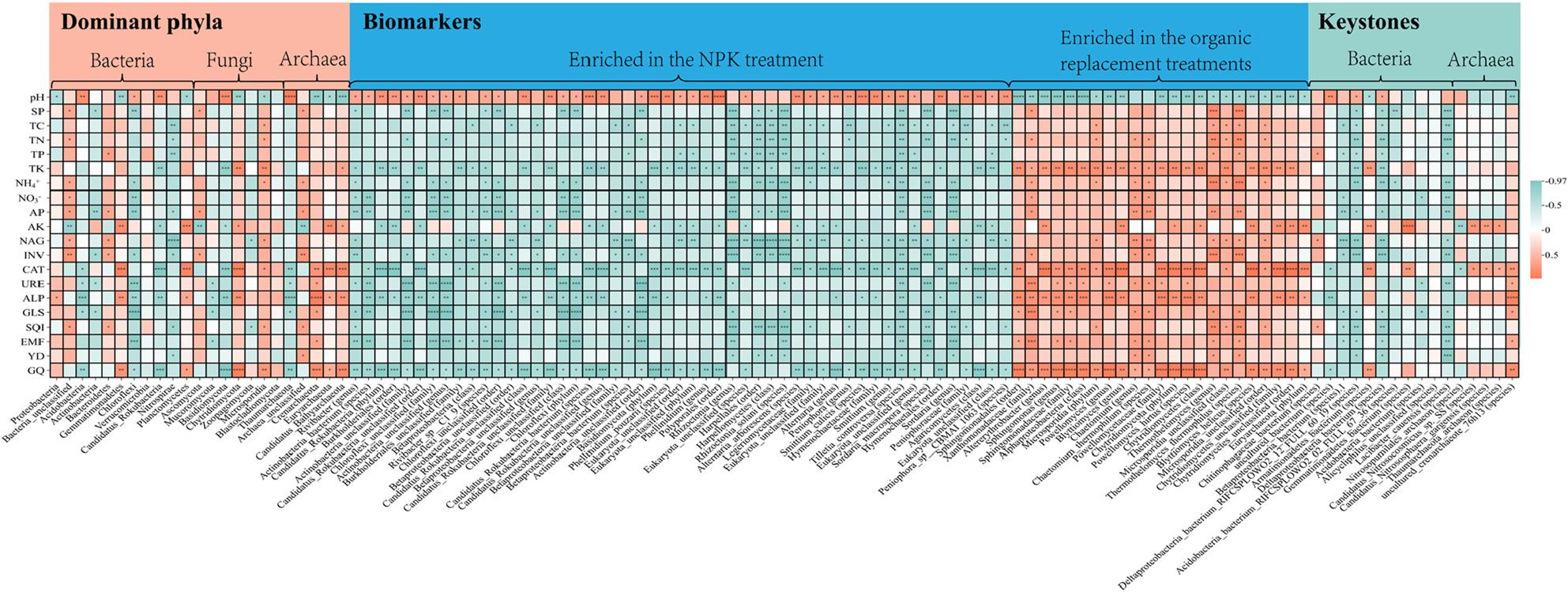
Spearman's correlation analysis heatmap of soil properties with dominant phyla, biomarkers, and keystones. Red and green indicate positive and negative correlations, respectively. * denotes *p* < 0.05, ** denotes *p* < 0.01, *** denotes *p* < 0.001 and **** denotes *p* < 0.0001. SP, soil total porosity; TC, total organic carbon; TN, total nitrogen; TP, total phosphorus; TK, total potassium; NH4+, ammonium nitrogen; NO3-, nitrate nitrogen; AP, available phosphorus; AK, available potassium; NAG, β-N-acetylglucosaminidase; INV, invertase; CAT, catalase; URE, urease; ALP, soil alkaline phosphatase; GLS, glutaminase; SQI, soil quality index; EMF, ecological multifunctionality; YD, yield; GQ, total flavonoid content

**Fig. 6:**
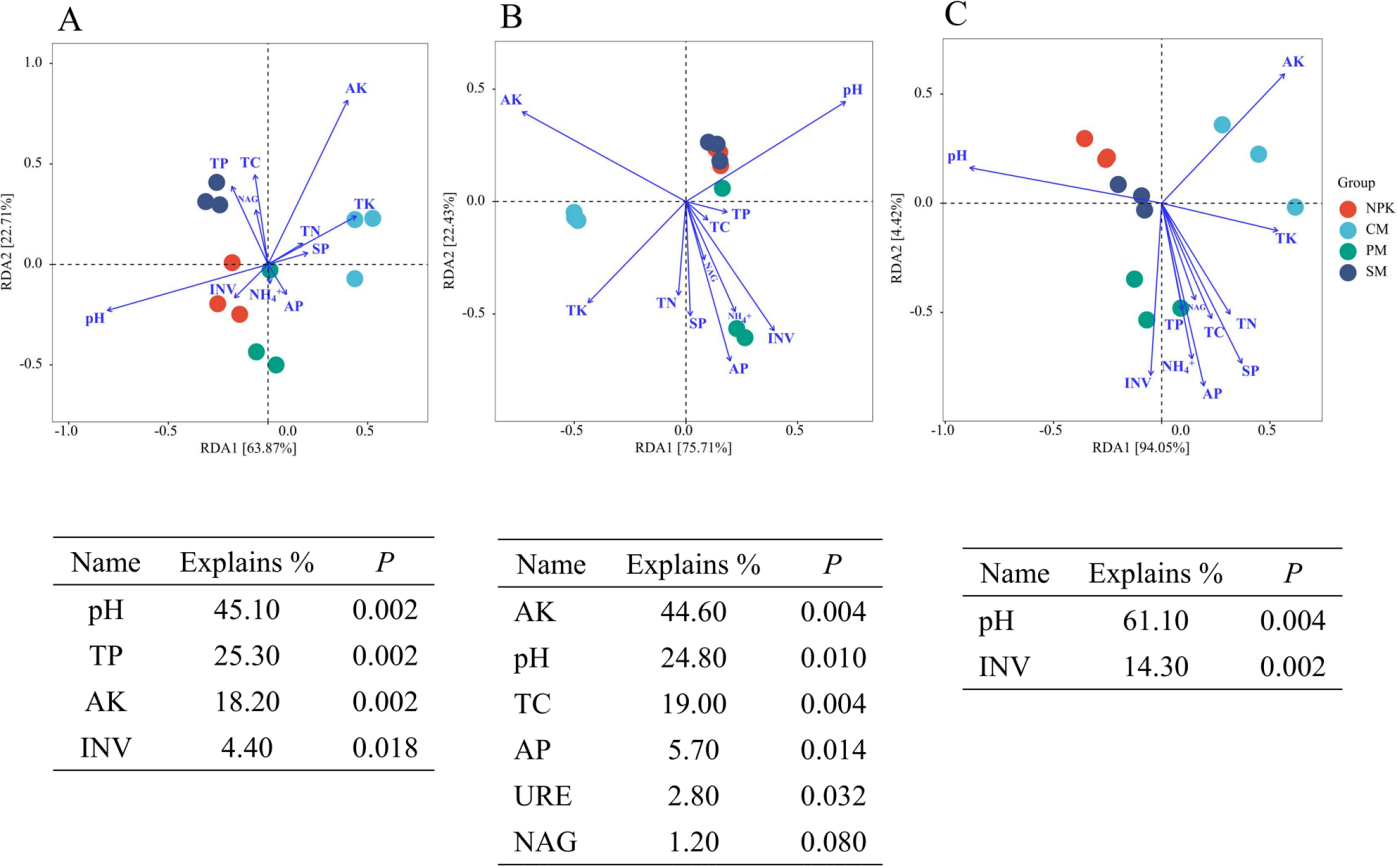
The relationships between dominant microbial phyla and soil environmental factors. **A**) Bacteria, (**B**) Fungi, (**C**) Archaea. Statistically significant environment factors and their explanations are shown below the plots. NPK: pure chemical fertilizer; CM: cow manure replacement; PM: pig manure replacement; SM: sheep manure replacement

Spearman’s correlation analysis revealed that Bacteroidetes and Ascomycota had negative correlations with TK and AK and positive correlations with most soil properties (Fig. 5). Acidobacteria, Chloroflexi, Basidiomycota, and Thaumarchaeota showed positive correlations with pH and negative correlations with most environmental factors, and the majority of these correlations were found to be significant (*p* < 0.05). The dominant archaeal phyla Crenarchaeota, Euryarchaeota, and Candidatus_Bathyarchaeota were positively correlated with CAT, ALP, and GQ (*p* < 0.05). The biomarkers enriched in the inorganic fertilizer treatment (NPK) were negatively correlated with all soil properties, excluding pH and AK, and most of these correlation coefficients were significant (*p* < 0.05). Conversely, the biomarkers identified in the partial organic replacement treatments (CM, PM, and SM) showed opposite trends. The majority of biomarkers identified in the partial organic replacement treatments exhibited significant negative associations with pH and significant positive associations with SQI, EMF, and GQ (*p* < 0.05). The keystone species *Armatimonadetes_bacterium* and *uncultured_crenarchaeote_76h13* exhibited significant negative correlations with pH (*p* < 0.05) and extremely significant positive correlations with CAT and GQ (*p* < 0.01). Moreover, redundancy analysis revealed that pH (explaining 45.10% of variance) had the most significant impact on bacterial community composition, followed by TP, AK, and INV (Fig. 6). AK, pH, and TC were three physicochemical indicators that had a significant effect on the fungal community (*p* < 0.01), among which AK had the most significant effect, explaining 44.60% of the variations in the fungal community. Both pH and INV significantly impacted the structure of the archaeal community at the phylum level (*p* < 0.01). In conclusion, pH was an important factor affecting soil microbial community structure.

### Key influencing factors of soil ecological multifunctionality

**Fig. 7:**
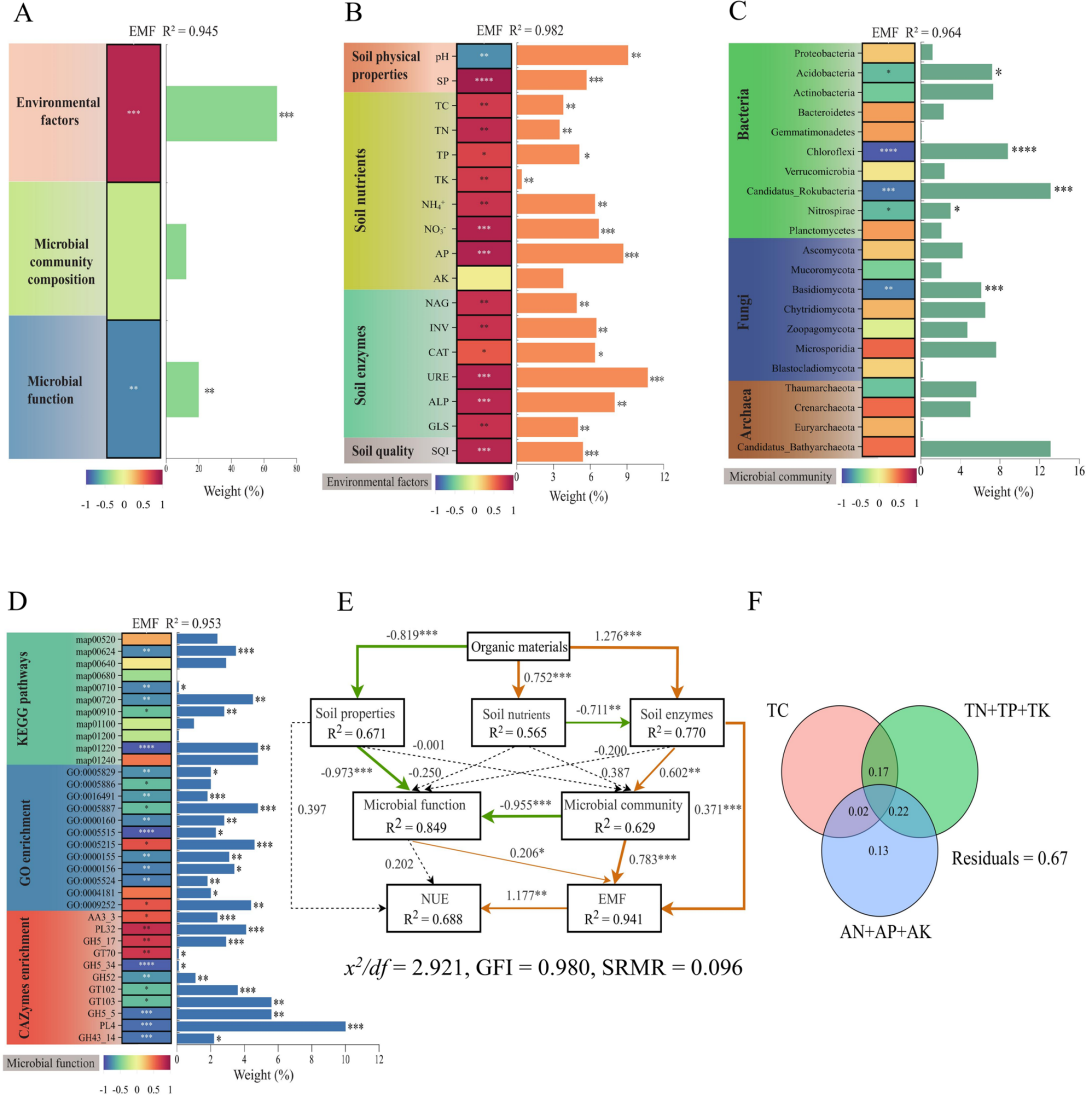
Key influencing factors of soil ecological multifunctionality (EMF). The importance of soil environmental factors, microbial community composition and microbial functions to EMF was evaluated using random forest regression, linear regression, and Pearson's correlation analysis (**A**-**D**). The partial least squares path model (PLS-PM) was constructed to analyze the direct and indirect contributions of soil physical properties, soil nutrients, soil enzyme activities, microbial communities, and microbial functions to EMF and N use efficiency (NUE; **E**). Standardized path coefficients are listed alongside each arrow, with orange, green, and black arrows representing positive, negative, and insignificant paths, respectively. Variance partitioning analysis (VPA) was employed to explore the explanation ratio of different soil nutrient factors to microbial variations (**F**). TC, total organic carbon; TN, total nitrogen; TP, total phosphorus; TK, total potassium; AN, average of ammonium nitrogen and nitrate nitrogen; AP, available phosphorus; AK, available potassium* denotes *p* < 0.05, ** denotes *p* < 0.01, and *** denotes *p* < 0.001

Random forest regression and Pearson’s correlation analysis were performed to examine the relationships among soil environmental factors, microbial community, microbial function, and EMF. Soil environmental factors were significantly associated with EMF (Fig. 7A), among which soil NO₃⁻, AP, and URE contents showed significant positive correlations with EMF (*p* < 0.05; Fig. 7B). Soil microbial functions, including transporter activity (GO:0005215), PL32, and PL4, were significantly related to EMF (Fig. 7D). In addition, microbial functional diversity indices (observed species and Chao1 index) exhibited significant positive correlations with EMF (Supplementary Fig. S7). Although the contribution of microbial community composition to EMF was relatively low, key taxa, including Acidobacteria, Chloroflexi, and Basidiomycota, showed significant negative correlations with EMF, suggesting a potential association between these taxa and EMF variation (Fig. 7A and C).

Organic materials were correlated with soil properties (λ = -0.819, *p* < 0.001), soil nutrients (λ = 0.752, *p* < 0.001), and soil enzymes (λ = 1.276, *p* < 0.001; Fig. 7E). Soil pH, which is part of soil properties, was significantly correlated with microbial function (λ = -0.973, *p* < 0.001). Soil nutrients were correlated with soil enzymes (λ = -0.711, *p* < 0.01), soil enzymes were correlated with the microbial community (λ = 0.602, *p* < 0.01), and the microbial community was correlated with microbial function (λ = -0.955, *p* < 0.001). Short-term partial organic fertilizer replacement improved soil environmental factors and regulated microbial community composition and function. These variables were directly correlated with EMF, which was positively correlated with crop NUE (λ = 1.177, *p* < 0.01; Fig. 7E). We further examined the relationships between soil nutrients and microbial communities. Variance partitioning analysis (VPA) showed that available nutrients (NH_4_^+^, NO_3_^−^, AP, and AK) explained the highest proportion of variation in microbial community composition, followed by total nutrients (TN, TP, and TK) and total carbon (TC) (Fig. 7F).

## Discussion

### Effects of partial organic fertilizer replacement on soil quality, crop yield, and grain quality

In this study, partial organic fertilizer replacement dramatically elevated soil organic matter, total nutrient, and available nutrient contents in foxtail millet fields (Table 1), consistent with and further elaborating on our prior discoveries [6]. Notably, this nutritional enhancement is closely associated with the decomposition dynamics of organic amendments, as manure decomposition over time directly mediates nutrient release rates and material stability [2, 27, 28]. Differences in soil nutrient levels among various animal manure replacement treatments are mainly due to the inherent characteristics and decomposition dynamics of the different organic fertilizers, with specific nutrient parameters peaking under distinct manure types (Table 1) — a finding aligning with previous studies [7, 12, 27, 28] and attributed to the slow, sustained nutrient release from manures. For instance, the higher TN, NH_₄_^⁺^, and NO_₃_^⁻^ in the PM treatment may be related to its higher nutrient mineralization rate and N release capacity, while the elevated TC in the SM treatment could stem from its higher recalcitrant carbon components and slower decomposition rate [29]. These differences are further regulated by enzyme activity changes during manure decomposition: higher protease and N metabolism activities in pig manure promote N decomposition, while stronger cellulase activities in sheep manure accelerate recalcitrant fiber degradation [2]. Additionally, Chukwuma et al. demonstrated that manure P release patterns are primarily determined by temporal changes in soil pH and organic C [30], and the relatively higher AP content in the PM treatment may be linked to soil pH adjustments driven by metabolites (e.g., organic acids) released during organic amendment decomposition, which neutralize soil alkalization [31]. Simultaneously, organic matter promotes soil aggregate formation, increasing soil SP [32], which, in turn, regulates the manure decomposition rate, reduces nutrient loss, and improves material stability [10, 33]. Critically, these improvements in soil physicochemical properties — driven by partial organic fertilizer replacement (the core complementarity concept of this study) — contributed to a notable increase in the soil quality index (Table 1). Soil extracellular enzymes primarily originate from soil microbial activity [3]. Following the application of organic fertilizers, the released nutrients and improved soil structure and texture provided the energies and suitable conditions for microbial growth, thereby spurring on latent enzyme activity (Table 1), which in turn facilitated the mineralization and nutrient release of organic matter, ultimately enhancing soil EMF [7]. The positive correlation between SQI and EMF further supported this view (Supplementary Fig. S2).

This study also found that the partial organic replacement treatments involving cow, pig and sheep manure significantly improved soil SQI and EMF compared to chemical fertilizer treatment, as well as increasing foxtail millet yield (Fig. 1). Of these, pig manure replacement demonstrated the most pronounced enhancement of both soil quality and grain yield, attributed to the more complete nutrient release during pig manure hydrolysis, which more effectively promoted soil available N and P than other manures [2, 34]. The slow, balanced nutrient release from microbially decomposed manure ensures alignment between crop nutrient demands throughout the growing season and soil supply, enhancing crop N use efficiency and yield [7] — supported by our findings of increased PEP_N_ and AE_N_ (Fig. 1). Moreover, the total flavonoid content under organic replacement treatments, particularly the cow replacement treatment, was notably higher than that under NPK treatment (Fig. 1), consistent with Sun et al. [35], which may be due to metabolites from organic amendment decomposition promoting crop secondary metabolism, similar to the regulatory effect of 67.84 g ha^− 1^ selenium fertilizer on Jingu 21’s flavonoid metabolism [36]. This highlights that organic replacement enables foxtail millet production in North China to improve grain quality without negatively impacting yield.

### Effects of partial organic fertilizer replacement on soil microbial community structure and function

The application of organic fertilizer to extensively cultivated agricultural soils enhances the ecological multifunctionality by improving soil microbial community structure and function, thereby accelerating nutrient cycling [4]. Our findings partial corroborated this discovery. Specifically, compared to NPK, different types of animal manure replacement treatments increased the observed species of soil bacteria, fungi, and archaea, along with the Chao1 and Shannon indexes (Supplementary Table 2), with the PM treatment exhibiting the highest bacterial observed species and Chao1 index. These variations may be attributed to higher soil aeration and nutrient availability under organic fertilization [18, 37]. Bacteria exhibit high sensitivity to the availability of C sources [18, 38, 39]. Compared to other manures, pig manure has a lower C-N ratio, requiring less energy for microbial decomposition, and thus it exhibited a higher mineralization rate and complete nutrient release, which may explain the significant increase in alpha diversity under PM treatment [27, 34, 40]. Notably, this increase in soil microbial diversity, driven by partial organic replacement, enhances soil ecological multifunctionality’s adaptive capacity to environmental shifts. Additionally, the PCoA results revealed notable differences in soil bacterial, fungal, and archaeal community structures between the NPK treatment and the partial organic replacement treatments (Fig. 2), consistent with the findings of Gao et al. [41]. CM, PM, and SM not only boosted soil microbial diversity but also altered community structure and composition relative to NPK (Supplementary Fig. S4). The RDA results further indicated that improved soil physical properties, increased nutrient levels, and enhanced enzyme activities played a key role in optimizing the microbial community structure, as evidenced by decreased soil pH, increased TC, TP, and AK levels, and enhanced INV and URE activities (Fig. 6).

Microorganisms prefer to coexist in large numbers under low nutrient conditions, whereas elevated nutrient availability drives a marked increase in negative interspecific interactions [42]. Applying organic fertilizer increased soil C and nutrient availability, reduced the proportion of positive edges and the network density of soil bacterial and fungal co-occurrence networks, thereby decreasing the complexity and enhancing network stability (Fig. 3, Supplementary Table 6). Conversely, under partial organic replacement treatments, the proportion of positive edges in soil archaeal networks increased, accompanied by reduced network complexity and stability. The finding aligns with those of Liu et al. [3], who observed that positive interactions continuously drove alterations and instability in the initial condition of ecosystems, while negative interactions regionally stabilize this process — collectively indicating that partial organic replacement enhances soil microbial network stability under external environmental perturbations. Additionally, we identified 30 keystones that maintain the stability and functional integrity of microbial networks, belonging to seven phyla (Fig. 3, Supplementary Table 7). Specifically, compared to NPK, the abundance of Proteobacteria, Gemmatimonadetes, Bacteroidetes, and Crenarchaeota was increased by partial organic replacement treatments, while the abundance of Acidobacteria, Armatimonadetes, and Thaumarchaeota was decreased (Supplementary Table 4). Notably, Proteobacteria, Gemmatimonadetes, Bacteroidetes, and Crenarchaeota phyla stabilized microbial networks as connectors and module hubs, exhibiting positive correlations with soil C, N, and P content and cycling enzyme activities, demonstrating characteristics of eutrophication (Supplementary Table 7, Supplementary Fig. S5). These eutrophic taxa play key roles in nutrient cycling: Proteobacteria and Bacteroidetes utilize C/N substances for rapid growth and drive C/N cycling [18, 43]; Gemmatimonadetes degrades insoluble phosphates via gcd and ppx genes [44]; and Crenarchaeota contribute to N cycling [45]. Partial organic replacement induces favorable soil environmental changes, with manure decomposition and mineralization providing nutrient-rich substrates that support the proliferation of these eutrophic taxa. In contrast, Acidobacteria, Armatimonadetes, and Thaumarchaeota exhibited oligotrophic characteristics, showing negative correlations with most soil environmental factors and lower competitiveness in nutrient-rich soils compared to eutrophic microorganisms [46]. Thus, partial organic fertilizer replacement enhances microbial community resilience by up-regulating eutrophic microbial abundance and down-regulating oligotrophic microbial abundance.

Many previous studies have indicated that among the known physicochemical properties associated with Acidobacteria in soil, pH is the most crucial [47, 48], and our findings corroborated this perspective. Furthermore, organic replacement treatments remarkably enriched 23 biomarkers, including *Alphaproteobacteria* (class), *Xanthomonadaceae* (family), Microsporidia (phylum), Chytridiomycota (phylum), Euryarchaeota (phylum), *Chaetomium* (genus), and *Thermothelomyces* (genus) (Supplementary Table 5). These biomarkers play functionally relevant roles in soil-plant systems: *Alphaproteobacteria* and Chytridiomycota decompose lignin and mineralize complex organic matter [49, 50]; *Xanthomonadaceae* suppresses wheat head blight and exerts biocontrol functions [51]; Euryarchaeota is linked to soil methane production [52]; and *Chaetomium* acts as an effective biofungicide for integrated plant disease management [53]. In summary, our findings suggest that foxtail millet can recruit beneficial microorganisms to optimize crop productivity depending on its nutritional requirements and soil fertility. The positive correlation between biomarkers enriched in organic replacement treatments and soil nutrients, SQI, EMF, YD, and GQ further supported this conclusion (Fig. 5). Notably, pig manure replacement resulted in a more pronounced increase in microbial diversity and enhanced network stability compared to cow and sheep manure, indicating that the application of pig manure establishes a more conducive soil environment for foxtail millet growth.

Importantly, partial organic fertilizer replacement indirectly improved soil microbial functions by changing microbial community structures and compositions (Fig. 4). Regarding soil KEGG metabolic pathways, organic partial replacement treatments enhanced microbial organic C catabolism relative to NPK, with increased soil organic matter activating C metabolism-related processes (e.g., starch and sucrose metabolism, amino sugar and nucleotide sugar metabolism, pentose phosphate pathway; Fig. 4D, Supplementary Table 8). Simultaneously, these treatments reduced C release pathways (e.g., methane metabolism, degradation of aromatic compounds), effectively retaining available C sources in the soil microenvironment and further boosting anabolic metabolism of certain polysaccharides and fatty acids, N metabolism, and biosynthesis of cofactors. This aligns with Qi et al. [54], who demonstrated that inhibiting aromatic compound degradation promotes efficient C sequestration. In terms of CAZymes metabolism, partial organic replacement promoted the metabolism of mannanase, β-porphyranase, and β-D-galactofuranosidase, whereas NPK enhanced xylosidase, endoglucanase metabolism, and α-mannosyltransferase synthesis (Fig. 4F, Supplementary Table 12). These CAZymes play functionally relevant roles: mannanase facilitates hemicellulose decomposition [55], β-porphyranase may be linked to plant disease resistance and microbial niche colonization [56], and β-D-galactofuranosidase hydrolyzes complex oligosaccharides [57]. These CAZymes variations, driven by partial organic replacement, contribute to improved soil quality and ecological multifunctionality. For GO enrichment analysis, CM, PM, and SM treatments significantly increased glycophagy, positive regulation of glycogen catabolic process, photosystem II assembly, N-acetylglucosamine biosynthetic process, positive regulation of RNA splicing, and negative regulation of glucokinase activity compared to NPK, promoting soil C/N metabolism and microbial homeostasis (Fig. 4E, Supplementary Table 9). Conversely, CM, PM, and SM treatments significantly decreased the activities of all-trans-beta-apo-10’-carotenal cleavage oxygenase and 9-cis-10’-apo-beta-carotenal cleavage oxygenase. The reduction in these enzyme activities leads to decreased catabolic metabolism of carotenoids. These changes coincided with alterations in CAZymes and KEGG pathways, further confirming that partial organic fertilizer replacement modulates soil enzyme activities to regulate microbial metabolic pathways, ultimately enhancing nutrient cycling.

### Effects of partial organic fertilizer replacement on soil ecological multifunctionality

The importance of organic fertilizers in balancing farmland ecosystems and enhancing soil quality has been underlined in prior research [31, 35]. This study confirmed that partial organic fertilizer replacement enhanced soil SQI and EMF (Table 1). Soil environmental factors and microbial function were key elements that contributed to EMF (Fig. 7A), with this study further revealing that soil NO_3_^−^, AP, and URE levels play a pivotal role in regulating EMF (Fig. 7B), aligning with the observations reported by Shi et al. [4]. Unlike chemical fertilizers that release nutrients rapidly, organic manures provide a sustained nutrient supply due to their slow‑release characteristics. The combined application of organic and chemical fertilizers can effectively improve the soil environment and ensure continuous nutrient availability throughout the grain-growing period [3, 4]. Notably, increased organic matter from partial organic replacement provides diverse ecological niches for microbial growth and reproduction [23]. Although the overall microbial community showed no significant response to EMF, key groups affected by EMF were identified: Acidobacteria (Connectors), Chloroflexi (Biomarkers), Nitrospirae, Candidatus_Rokubacteria (Biomarkers), and Basidiomycota (Biomarkers) (Fig. 7A and C). These groups play vital roles in maintaining the structure of microbial communities as network connectors or shared biomarkers. Additionally, they harbor genes involved in C cycling (e.g., CAZyme family genes and Wood-Ljungdahl pathway-related genes), N cycling (e.g., *amo*, and *hao*), and P cycling (e.g., *pho2*, *pho80*, and *pho81*), demonstrating varied metabolic abilities and broad ecological adaptability [58–60]. Additionally, increased microbial diversity under partial organic replacement treatments also improved soil EMF (Supplementary Fig. S7). Correlation analysis revealed a positive association between soil C and N cycling processes and EMF (Fig. 7D), indicating these biochemical processes play a crucial role in regulating EMF. In summary, partial organic fertilizer replacement increases soil organic matter and nutrient content, thereby elevating the relative abundance of beneficial soil microorganisms. This promotes soil nutrient cycling, boosts the supply of available nutrients and enzyme activity, and ultimately establishes a positive feedback mechanism for EMF. This reinforces the value of complementarity-driven organic replacement rather than mere organic fertilizer application in optimizing farmland ecosystem balance and soil quality.

In view of the critical importance of soil environment factors and microorganisms in EMF, our research further analyzed the correlation between soil environmental factors and microorganisms, as well as their contributions to EMF and NUE. PLS-PM results revealed that partial organic fertilizers enhanced soil EMF through two complementary mechanisms: (1) directly, by altering soil nutrient levels and enzyme activities, and (2) indirectly, by improving soil microbial community diversity and function, thereby enhancing the soil EMF (Fig. 7E). Importantly, partial organic replacement also enhanced foxtail millet NUE by improving soil EMF, with improved soil environmental factors indirectly modulating microbial functions via shifts in microbial community structure. Consistent with this, RDA results indicated that pH was a key factor influencing the distribution of soil microbial unigenes (Fig. 6). Similar results were found by Han et al. and Bahram et al. [61, 62]. Furthermore, the correlation analysis revealed that most environmental factors showed significant positive correlations with biomarkers enriched in partial organic replacement treatments and significant negative correlations with biomarkers enriched in the NPK treatment (Fig. 5), with pH showing an opposite trend to other physicochemical properties. Collectively, these results indicate that partial organic replacement is strongly associated with changes in soil nutrient levels and microbial communities, which jointly promote increased foxtail millet NUE and yield.

In summary, short-term partial organic replacement, particularly 50% partial pig manure replacement, is a feasible strategy to enhance foxtail millet NUE and grain quality by regulating soil nutrients and microbial communities. Notably, the soil heavy metal pollution index is also a crucial indicator of soil health, and attention must be paid to the risks of soil heavy metal pollution under long-term organic replacement. Future research should focus on the long-term effects of partial organic replacement on soil health, microbial function, greenhouse gas emissions, and economic viability, based on the temporal decomposition characteristics of organic amendments. This research is crucial for mitigating environmental pollution caused by livestock manure, as well as for addressing the decline in crop productivity and the severe disruption of farmland ecosystems resulting from the excessive application of synthetic fertilizers.

## Conclusion

This study investigated soil microbial communities and their relationships with environmental factors under different animal manure replacement treatments. The results demonstrated that partial organic fertilizer replacement, especially pig manure, significantly increased soil nutrient levels and enzyme activities, reduced soil alkalinity, and directly improved soil EMF in foxtail millet fields. The improved soil environment optimized microbial community structure and composition, regulated the abundance of enzymes involved in carbon and N metabolism, and enhanced related microbial metabolic pathways, further indirectly contributing to higher soil EMF. Partial organic replacement also mediated the associations between soil microbial communities and environmental factors, thus regulating soil microecology, with pH as the dominant driving factor. Key microbial phyla, such as Proteobacteria, played a critical role in maintaining network stability and functional performance. Overall, partial organic replacement enhanced soil EMF by improving soil properties and regulating microbial community structure and function, thereby increasing crop N use efficiency and grain yield, with partial pig manure replacement showing the most significant effect. Future studies should focus on long-term monitoring of greenhouse gas emissions, crop yield stability, soil health succession, microbial community dynamics, and economic feasibility for farmers, to provide a comprehensive basis for sustainable fertilization strategies.

## Supplementary Information

Supplementary Material 1.

Supplementary Material 2.

## Data Availability

The datasets generated during the current study are available in the NCBI Sequence Read Archive (SRA) repository under the BioProject accession number PRJNA1373209 (https://www.ncbi.nlm.nih.gov/sra/PRJNA1373209).

## References

[1] Li HY, Wei ZM, Song CH, Chen XM, Zhang RJ, Liu YM. Functional keystone drive nitrogen conversion during different animal manures composting. Bioresource technol. 2022;361:127721. 10.1016/J.BIORTECH.2022.127721. 10.1016/j.biortech.2022.127721 35914672

[2] Cheng YR, Wan WJ. Alkaline phosphomonoesterase-harboring bacteria facilitate phosphorus availability during winter composting with different animal manures. J Clean Prod. 2022;376:134299. 10.1016/J.JCLEPRO.2022.134299.

[3] Liu JA, Shu AP, Song WF, Shi WC, Li MC, Zhang WX, Li ZZ, Liu GR, Yuan FS, Zhang SX, Liu ZB, Gao Z. Long-term organic fertilizer substitution increases rice yield by improving soil properties and regulating soil bacteria. Geoderma. 2021;404:115287. 10.1016/J.GEODERMA.2021.115287.

[4] Shi YL, Li TT, Zheng L, Jing XK, Hussain HA, Zhang QW. Enhancing soil multifunctionality through restoring erosion environment and microbial functions combined with organic manure and straw mulching. Agr Ecosyst Environ. 2025;383:109515–109515. 10.1016/J.AGEE.2025.109515.

[5] Tang Q, Cotton A, Wei ZJ, Xia YQ, Daniell T, Yan XY. How does partial substitution of chemical fertilizer with organic forms increase sustainability of agricultural production? Sci. Total Environ. 2021;803:149933. 10.1016/J.SCITOTENV.2021.149933. 10.1016/j.scitotenv.2021.149933 34482141

[6] Nie ME, Gao X, Zhao LJ, Han GL, Duan YY, Han RH, Dong SQ, Li YL, Du HL, Yuan XY, Yang YJ. Organic substitution enhances soil quality, soil microbial community stability, foxtail millet productivity, and grain quality in North China. J Environ Manage. 2025;384:125613. 10.1016/J.JENVMAN.2025.125613. 40318616 10.1016/j.jenvman.2025.125613

[7] Wu G, Huang HM, Jia BB, Hu LL, Luan CS, Wu Q, Wang XY, Li XX, Che Z, Dong ZR, Song H. Partial organic substitution increases soil quality and crop yields but promotes global warming potential in a wheat-maize rotation system in China. Soil Tillage Res. 2024;244:106274. 10.1016/J.STILL.2024.106274.

[8] Li P, Li YB, Xu LY, Zhang HJ, Shen XS, Xu HF, Hu F. Crop yield-soil quality balance in double cropping in China’s upland by organic amendments: A meta-analysis. Geoderma. 2021;403:115197. 10.1016/J.GEODERMA.2021.115197.

[9] Abbas A, Naveed M, Shehzad Khan K, Ashraf M, Siddiqui MH, Abbas N, Mutafa A, Ali L. The efficacy of organic amendments on maize productivity, soil properties and active fractions of soil carbon in organic-matter deficient soil. Span J Soil Sci. 2024;14:12814. 10.3389/sjss.2024.12814.

[10] Sihi D, Dari B, Sharma D, Pathak H, Nain L, Sharma OP. Evaluation of soil health in organic vs. conventional farming of basmati rice in North India. J Plant Nutr Soil Sci. 2017;180:389–406. 10.1002/jpln.201700128.

[11] Wang XY, Zhao SC, Xu XP, Liu MJ, Jiang R, Zhang J, Duan Y, He P, Zhou W. Response of soil microbial properties in the life cycle of potatoes to organic substitution regimes in North China. Soil Tillage Res. 2024;237:106000. 10.1016/J.STILL.2024.106000.

[12] Ma L, Li ZS, Li Y, Wei JL, Zhang LF, Zheng FL, Liu ZH, Tan DS. Variations in crop yield caused by different ratios of organic substitution are closely related to microbial ecological clusters in a fluvo-aquic soil. Field Crop Res. 2024;306:109239. 10.1016/J.FCR.2023.109239.

[13] Hu XJ, Gu HD, Liu JJ, Wei D, Zhu P, Cui XA, Zhou BK, Chen XL, Jin J, Liu XB, Wang GH. Metagenomics reveals divergent functional profiles of soil carbon and nitrogen cycling under long-term addition of chemical and organic fertilizers in the black soil region. Geoderma. 2022;418:115846. 10.1016/J.GEODERMA.2022.115846.

[14] Li GC, Niu WQ, Ma L, Du YD, Zhang Q, Sun J, Siddique Kadambot HM. Legacy effects of wheat season organic fertilizer addition on microbial co-occurrence networks, soil function, and yield of the subsequent maize season in a wheat-maize rotation system. J Environ Manage. 2023;347:119160. 10.1016/J.JENVMAN.2023.119160. 37812905 10.1016/j.jenvman.2023.119160

[15] Liu M, Song F, Yin ZH, Chen P, Zhang ZX, Qi ZJ, Wang B, Zheng EN. Organic fertilizer substitutions maintain maize yield and mitigate ammonia emissions but increase nitrous oxide emissions. Environ Sci Pollut Res. 2023;30:53115–27. 10.1007/S11356-023-25666-6. 10.1007/s11356-023-25666-6 36853529

[16] The FAOSTAT Database. Foxtail millet production and area harvested. Food and Agriculture Organization of the United Nations. https://www.fao.org/faostat/en/#data/QCL. Accessed 5 Mar 2013.

[17] Han GL, Wang J, Zhao HY, Wang D, Duan YY, Han RH, Nie ME, Zhao LJ, Du HL. Response of quality and yield of foxtail millet to nitrogen and Zinc application. Agriculture. 2023;13(9):1731. 10.3390/AGRICULTURE13091731.

[18] Ren JH, Liu XL, Yang WP, Yang XX, Li WG, Xia Q, Li JH, Gao ZQ, Yang ZP. Rhizosphere soil properties, microbial community, and enzyme activities: Short-term responses to partial substitution of chemical fertilizer with organic manure. J Environ Manage. 2021;299:113650. 10.1016/J.JENVMAN.2021.113650. 34481370 10.1016/j.jenvman.2021.113650

[19] Ma K. Effects of the ecological factors in different production areas on the grain quality of Jingu21. Shanxi Agricultural Univ. 2022. 10.27285/d.cnki.gsxnu.2022.000398.

[20] Bao SD. Soil Agrochemical Analysis. 3rd ed. Beijing: China Agricultural; 2020.

[21] Guan SY. Soil enzymes and their research methods. 1st ed. Beijing: China Agricultural; 1986.

[22] Cao XC, Liu L, Ma QX, Lu RH, Kong HM, Kong YL, Zhu LF, Zhu CQ, Tian WH, Jin QY, Wu LH, Zhang JH. Optimum organic fertilization enhances rice productivity and ecological multifunctionality via regulating soil microbial diversity in a double rice cropping system. Field Crop Res. 2024;318:109569. 10.1016/J.FCR.2024.109569.

[23] Delgado-Baquerizo M, Maestre FT, Reich PB, Jeffries TC, Gaitan JJ, Encinar D, Berdugo M, Campbell CD, Singh BK. Microbial diversity drives multifunctionality in terrestrial ecosystems. Nat commun. 2016;7(1):10541. 10.1038/ncomms10541. 26817514 10.1038/ncomms10541 PMC4738359

[24] Nabiollahi K, Golmohamadi F, Taghizadeh-Mehrjardi R, Kerry R, Davari M. Assessing the effect of slope gradient and land use change on soil quality degradation through digital mapping of soil quality indices and soil loss rate. Geoderma. 2018;318:16–28. 10.1016/j.geoderma.2017.12.024.

[25] Kuzyakov Y, Gunina A, Zamanian K, Tian J, Luo Y, Xu XL, Yudina A, Aponte H, Alharbi H, Ovsepyan L, Kurganova I, Ge T, Guillaume T. New approaches for evaluation of soil health, sensitivity and resistance to degradation. Front Agr Sci Eng. 2020;7(3):282–8. 10.15302/j-fase-2020338.

[26] Cheng Y, Qiao RN, Ding YT, Dong QG, Feng H, Zhang TB. Effects of chemical fertilizer reduction and organic substitution on water and salt characteristics of high salinity soil and water and nitrogen use efficiency of sunflower. Plant Nutr Fert Sci. 2021;27(11):1981–92. 10.11674/zwyf.2021173.

[27] Ruangcharus C, Un Kim S, Yoo G, Choi E, Kumar S, Kang N, Oh Hong C. Nitrous oxide emission and sweet potato yield in upland soil: Effects of different type and application rate of composted animal manures. Environ Pollut. 2021;279:116892–116892. 10.1016/J.ENVPOL.2021.116892. 33751943 10.1016/j.envpol.2021.116892

[28] Khan KS, Ali MM, Naveed M, Rehmani MIA, Shafique MW, Ali HM, Abdelsalam NR, Ghareeb RY, Feng G. Co-application of organic amendments and inorganic P increase maize growth and soil carbon, phosphorus availability in calcareous soil. Front Environ Sci. 2022;10:949371. 10.3389/fenvs.2022.949371.

[29] Wan JX, Wang XF, Yang TJ, Wei Z, Banerjee S, Friman V, Mei XL, Xu YC, Shen QR. Livestock manure type affects microbial community composition and assembly during composting. Front Microbiol. 2021;12:621126–621126. 10.3389/FMICB.2021.621126. 33828537 10.3389/fmicb.2021.621126 PMC8019744

[30] Chukwuma CC, Oraegbunam CJ, Ndzeshala SD, Uchida Y, Ugwu VU, Obalum SE, Igwe CA. Phosphorus mineralization in two lithologically dissimilar tropical soils as influenced by animal manure type and amendment-to-sampling time interval. Commun Soil Sci Plan. 2024;55(5):707–22. 10.1080/00103624.2023.2276269.

[31] Nie ME, Yue GQ, Wang L, Zhang YZ. Short-term organic fertilizer substitution increases sorghum yield by improving soil physicochemical characteristics and regulating microbial community structure. Front Plant Sci. 2024;15:1492797. 10.3389/FPLS.2024.1492797. 39582622 10.3389/fpls.2024.1492797 PMC11581943

[32] Feng HL, Han XZ, Biswas A, Zhang M, Zhu YC, Ji YX, Lu XC, Chen X, Yan J, Zou WX. Long-term organic material application enhances black soil productivity by improving aggregate stability and dissolved organic matter dynamics. Field Crop Res. 2025;328:109946–109946. 10.1016/J.FCR.2025.109946.

[33] Raza T, Qadir MF, Khan KS, Eash NS, Yousuf M, Chatterjee S, Manzoor R, Rehman S, Oetting JN. Unraveling the potential of microbes in decomposition of organic matter and release of carbon in the ecosystem. J Environ Manage. 2023;344:118529. 10.1016/j.jenvman.2023.118529. 37418912 10.1016/j.jenvman.2023.118529

[34] Jia ZY, Liu Y, Zhao SQ, Li XT, Li ZY. Effects of partial substitution of chemical fertilizers with different manures on soil properties and vegetable nitrogen utilization in facility vegetable plots. J Water Irrig. 2025;07:31–8. 10.12396/jsgg.2025013.

[35] Sun MJ, Chao Y, He W, Kang XR, Yang QG, Wang H, Pan H, Lou YH, Zhuge YP. Changes in foxtail millet (*Setaria italica* L.) yield, quality, and soil microbiome after replacing chemical nitrogen fertilizers with organic fertilizers. Sustainability. 2022;14(24):16412–16412. 10.3390/SU142416412.

[36] Wang S, Mao J, Xu Y, Liu S, Qiao Z, Cao X. Optimal selenium fertilizer affects the formation of foxtail millet (*Setaria italica* L.) quality by regulating flavonoid metabolism and amino acid metabolism. Food Sci Nutr. 2025;1370362. 10.1002/fsn3.70362. 10.1002/fsn3.70362 PMC12124234 40452795

[37] Han Y, Ma ZL, Chen R, Wen Y, Liang YH, Zhang JZ, Li WH, Wang ZH. Organic substitution regulated the soil microbial communities by modifying nutrient limitations in saline-alkaline soils. Agr Water Manage. 2025;316:109586–109586. 10.1016/J.AGWAT.2025.109586.

[38] Liu QX, Wan XJ, Chen HX, Wang JS, Sun AQ, Dong EW, Wang Y, Huang XL, Huang WH, Jiao XY. Organic fertilization enhances temporal stability of rhizosophere soil microbiomes in a long-term sorghum field experiment. J Soils Sediments. 2025;25:650–61. 10.1007/s11368-025-03975-2.

[39] Wen L, Luo HF, Li C, Cheng KK, Shi L, Liu LH, Wang K, Tang HM. Substituting chemical by organic fertilizer improves soil quality, regulates the soil microbiota and increases yields in *Camellia oleifera*. Microorganisms. 2025;13:2509. 10.3390/microorganisms13112509. 41304195 10.3390/microorganisms13112509 PMC12654687

[40] Hu N, Wang X, Pang L, Lu J, Yang J, Xiao X, Khan KS. Ecological trade-offs of plastic film and straw mulching: mechanistic insights from soil structure and carbon–nitrogen. Agronomy. 2026;16(4):470. 10.3390/AGRONOMY16040470.

[41] Gao ZQ, Wang XT, Ding XW, Gao X, Han YN, Gong B, Wang J, Li WQ, Wu FH. Bio-organic fertilizers containing potential biocontrol strains suppress bacterial soft rot and reshape soil microbial communities in cucumbers. Biol Control. 2025;210:105891–105891. 10.1016/J.BIOCONTROL.2025.105891.

[42] Zhu PP, Liu CY, Wei W, Wu YX, Sardar MF, Yu XN, Guo WH. Stochastic processes limit the effect of organic fertilizer application on soil bacterial community composition in salt marsh *Suaeda salsa*. J Clean Prod. 2024;441:141034. 10.1016/J.JCLEPRO.2024.141034.

[43] Peng YH, Cui KP, Jian HM, Zhang Z, Chen LS, Xu YM, Li ZG, Liu HS, Xu T, Wang R. Synergistic effects of different endophytic Actinobacteria combined with organic fertilizer on soil nutrients and microbial diversity in *camellia oleifera*. Microorganisms. 2025;13(6):1396–1396. 10.3390/MICROORGANISMS13061396. 40572284 10.3390/microorganisms13061396 PMC12195596

[44] Singh M, Tejo Prakash T. Characterisation of phosphate solubilising bacteria in sandy loam soil under chickpea cropping system. Indian J microbiol. 2012;52(2):167–73. 10.1007/s12088-011-0209-z. 23729877 10.1007/s12088-011-0209-z PMC3386451

[45] Nicol GW, Schleper C. Ammonia-oxidising Crenarchaeota: important players in the nitrogen cycle? Trends Microbiol. 2006;14(5):207–12. 10.1016/j.tim.2006.03.004. 16603359 10.1016/j.tim.2006.03.004

[46] Yang Y, Dou YX, Wang BR, Xue ZJ, Wang YQ, An SS, Chang Scott X. Deciphering factors driving soil microbial life-history strategies in restored grasslands. iMeta. 2022;2:66. 10.1002/IMT2.66. 10.1002/imt2.66 PMC10989924 38868332

[47] Lian JS, Wang HY, Deng Y, Xu MG, Liu ST, Zhou BK, Jangid K, Duan YH. Impact of long-term application of manure and inorganic fertilizers on common soil bacteria in different soil types. Agr Ecosyst Environ. 2022;337:108044. 10.1016/J.AGEE.2022.108044.

[48] Dang PF, Li CF, Lu C, Zhang MM, Huang TT, Wan CX, Wang HY, Chen YL, Qin XL, Liao YC, Siddique Kadambot HM. Effect of fertilizer management on the soil bacterial community in agroecosystems across the globe. Agr Ecosyst Environ. 2022;326:107795. 10.1016/J.AGEE.2021.107795.

[49] Yu TB, Jie XT, Lei YG, Zhang BW, Zang HD, Zeng ZH, Yang YD. Rhizobacteria shaped by long-term fertilization and wheat nutritional requirements improve grain yield and soil ecological multifunctionality. Field Crop Res. 2025;333:110117–110117. 10.1016/J.FCR.2025.110117.

[50] Azeem M, Wang J, Kubwimana JJ, Kazmi SSH, Khan ZH, He KW, Han RX. Biochar-derived dissolved organic matter (BDOM) shifts fungal community composition: BDOM-soil DOM interaction. Appl Soil Ecol. 2025;207:105916–105916. 10.1016/J.APSOIL.2025.105916.

[51] Campos SB, Lisboa BB, Camargo FAO, Bayer C, Sczyrba A, Dirksen P, Albersmeier A, Kalinowski J, Beneduzi A, Costa PB, Passaglia LMP, Vargas LK, Wendisch VF. Soil suppressiveness and its relations with the microbial community in a Brazilian subtropical agroecosystem under different management systems. Soil Biol Biochem. 2016;96:191–7. 10.1016/j.soilbio.2016.02.010.

[52] Xu ZC, Li GX, Huda N, Zhang BX, Wang M, Luo WH. Effects of moisture and carbon/nitrogen ratio on gaseous emissions and maturity during direct composting of cornstalks used for filtration of anaerobically digested manure centrate. Bioresource Technol. 2020;298:122503. 10.1016/j.biortech.2019.122503. 10.1016/j.biortech.2019.122503 31837581

[53] Lavanya S, Sudha A, Karthikeyan G, Swarnakumari N. Priyank Hanuman Mhatre, Bharathi N. *Chaetomium* spp.: A multifaceted fungal biocontrol agent for sustainable management of crop diseases. Physiol Mol Plant P. 2025;140:102939–102939. 10.1016/J.PMPP.2025.102939.

[54] Qi JY, Yao XB, Lu J, He LX, Cao JL, Kan ZR, Wang X, Pan SG, Tang XR. A 40% paddy surface soil organic carbon increase after 5-year no-tillage is linked with shifts in soil bacterial composition and functions. Sci Total Environ. 2023;859:160206–160206. 10.1016/J.SCITOTENV.2022.160206. 36400297 10.1016/j.scitotenv.2022.160206

[55] Guo WW, Hui LF, Song FF, Qu Y, Wang QS, Zhang YY, Xin JT, Zhang TT. A new strategy for biological enzyme bleaching: combined effects of laccase, xylanase, and mannanase in the bleaching of softwood kraft pulp – a synergistic effect of enzymes. Nord Pulp Pap Res J. 2025;40(3):465–76. 10.1515/NPPRJ-2025-0017.

[56] Manat G, Fanuel M, Jouanneau D, Jam M, Mac-Bear J, Rogniaux H, Mora T, Larocque R, Lipinska A, Czjzek M, Ropartz D, Ficko-Blean E. Specificity of a β-porphyranase produced by the carrageenophyte red alga *Chondrus crispus* and implications of this unexpected activity on red algal biology. J biol chem. 2022;298(12):102707–102707. 10.1016/J.JBC.2022.102707. 36402445 10.1016/j.jbc.2022.102707 PMC9771727

[57] Matsunaga E, Tanaka Y, Toyota S, Yamada H, Oka H, Higuchi Y, Takegawa K. Identification and characterization of β-D-galactofuranosidase from *Aspergillus nidulans* and *Aspergillus fumigatus*. J Biosci Bioeng. 2020;131(1):1–7. 10.1016/j.jbiosc.2020.09.006. 33011078 10.1016/j.jbiosc.2020.09.006

[58] Daims H, Lebedeva EV, Pjevac P, Han P, Herbold C, Albertsen M, Jehmlich N, Palatinszky M, Vierheilig J, Bulaev A, Kirkegaard RH, von Bergen M, Rattei T, Bendinger B, Nielsen PH, Wagner M. Complete nitrification by *Nitrospira bacteria*. Nature. 2015;528(7583):504–9. 10.1038/nature16461. 26610024 10.1038/nature16461 PMC5152751

[59] Diamond S, Andeer PF, Li Z, Crits-Christoph A, Burstein D, Anantharaman K, Lane KR, Thomas BC, Pan C, Northen TR, Banfield JF. Mediterranean grassland soil C-N compound turnover is dependent on rainfall and depth, and is mediated by genomically divergent microorganisms. Nat Microbiol. 2019;4(8):1356–67. 10.1038/s41564-019-0449-y. 31110364 10.1038/s41564-019-0449-y PMC6784897

[60] Sewell HL, Kaster A, Spormann AM. Homoacetogenesis in deep-sea *Chloroflexi*, as inferred by single-cell genomics, provides a link to reductive dehalogenation. Terr Dehalococcoidetes mBio. 2017;8(6):e02022–17. 10.1128/mBio.02022-17. 10.1128/mBio.02022-17 PMC5736913 29259088

[61] Han ZQ, Xu PS, Li ZT, Lin HY, Zhu C, Wang JY, Zou JW. Microbial diversity and the abundance of keystone species drive the response of soil ecological multifunctionality to organic substitution and biochar amendment in a tea plantation. GCB Bioenergy. 2022;14(4):481–95. 10.1111/GCBB.12926.

[62] Bahram M, Hildebrand F, Forslund SK, Anderson JL, Soudzilovskaia NA, Bodegom PM, Bengtsson-Palme J, Anslan S, Coelho LP, Harend H, Huerta-Cepas J, Medema MH, Maltz MR, Mundra S, Olsson PA, Pent M, Põlme S, Sunagawa S, Ryberg M, Tedersoo L, Bork P. Structure and function of the global topsoil microbiome. Nature. 2018;560(7717):233–7. 10.1038/s41586-018-0386-6. 30069051 10.1038/s41586-018-0386-6

