# Supplementary material for: Organic amendments as partial replacements for synthetic fertilizers in foxtail millet
Source: BMC Plant Biol. 2026 Apr 7;26:864. doi: 10.1186/s12870-026-08660-1 (PMC13181885; doi:10.1186/s12870-026-08660-1)
Supplement: Supplementary file 2 — Supplementary Material 2. [file 12870_2026_8660_MOESM2_ESM.docx]

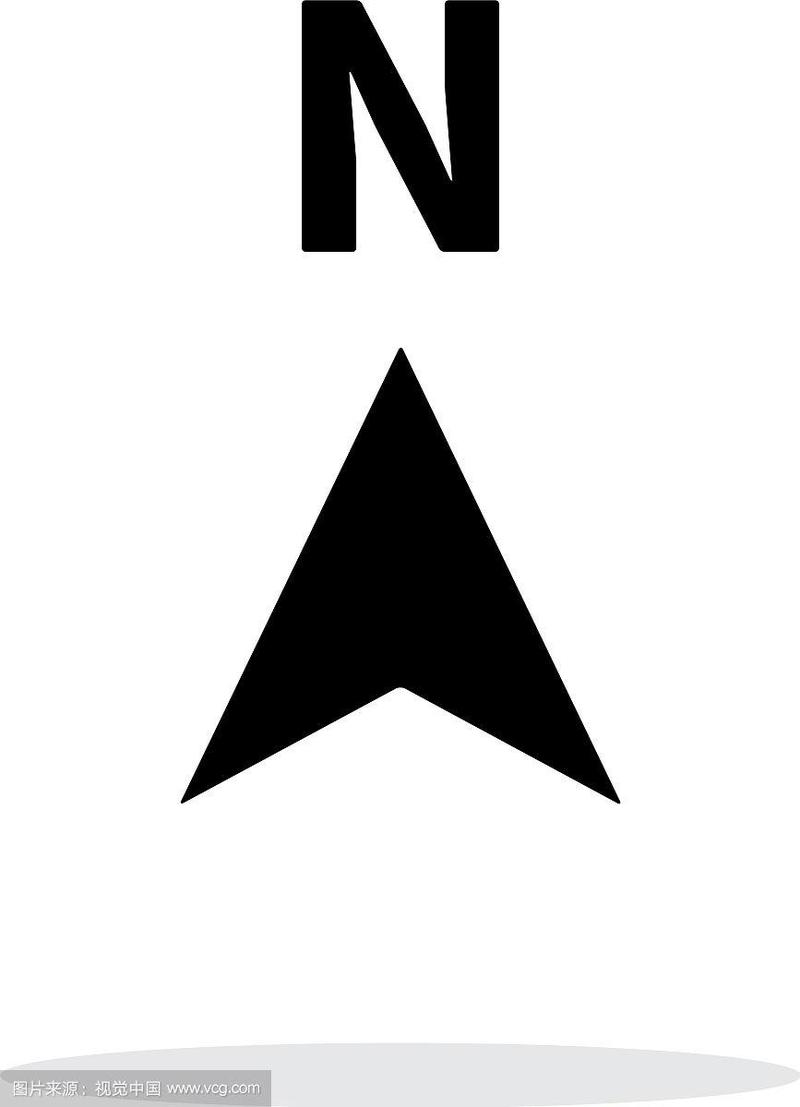

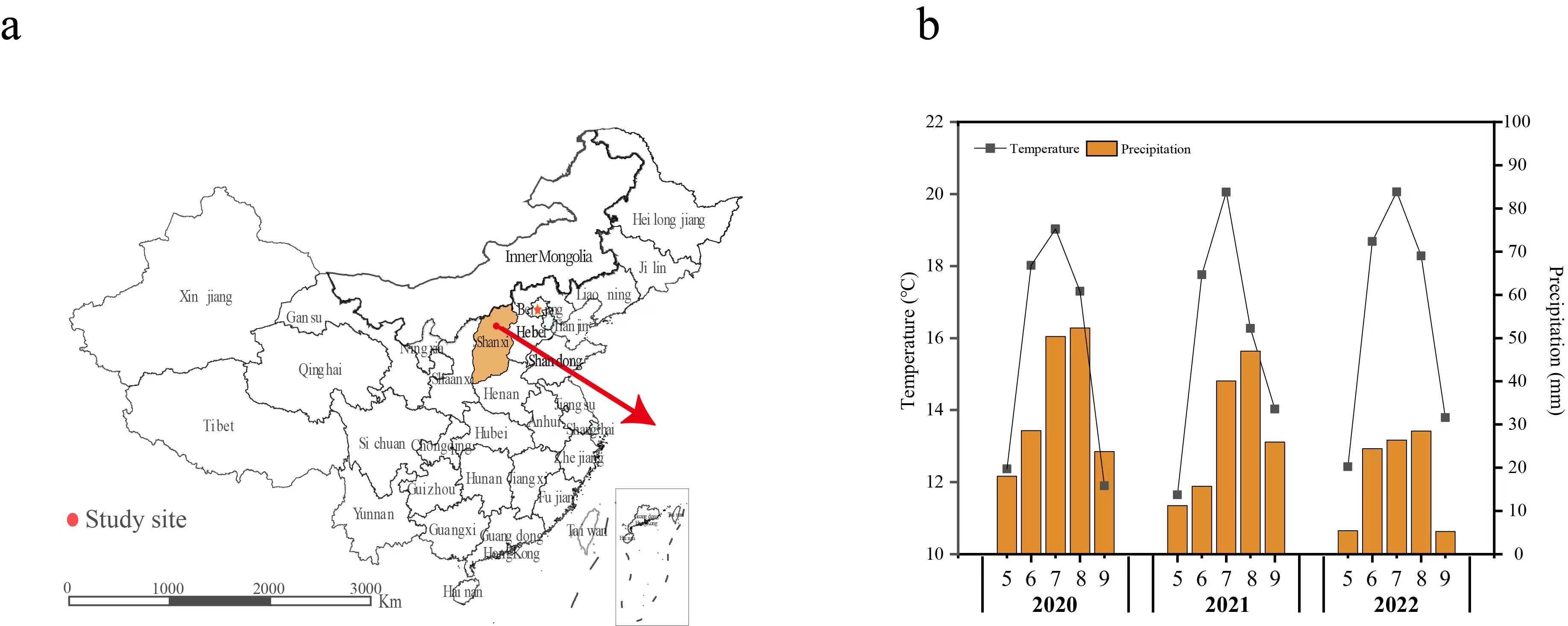


A


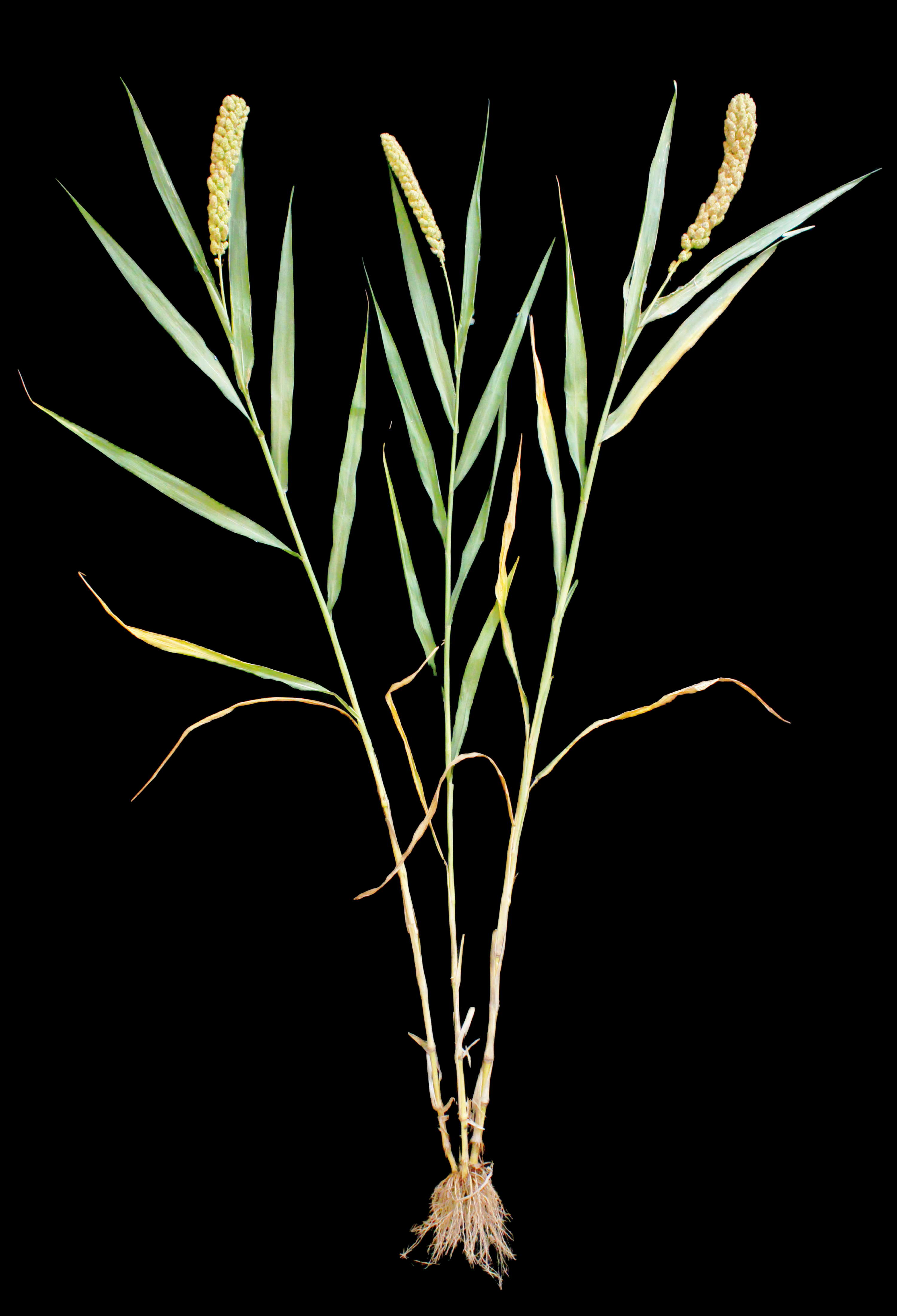

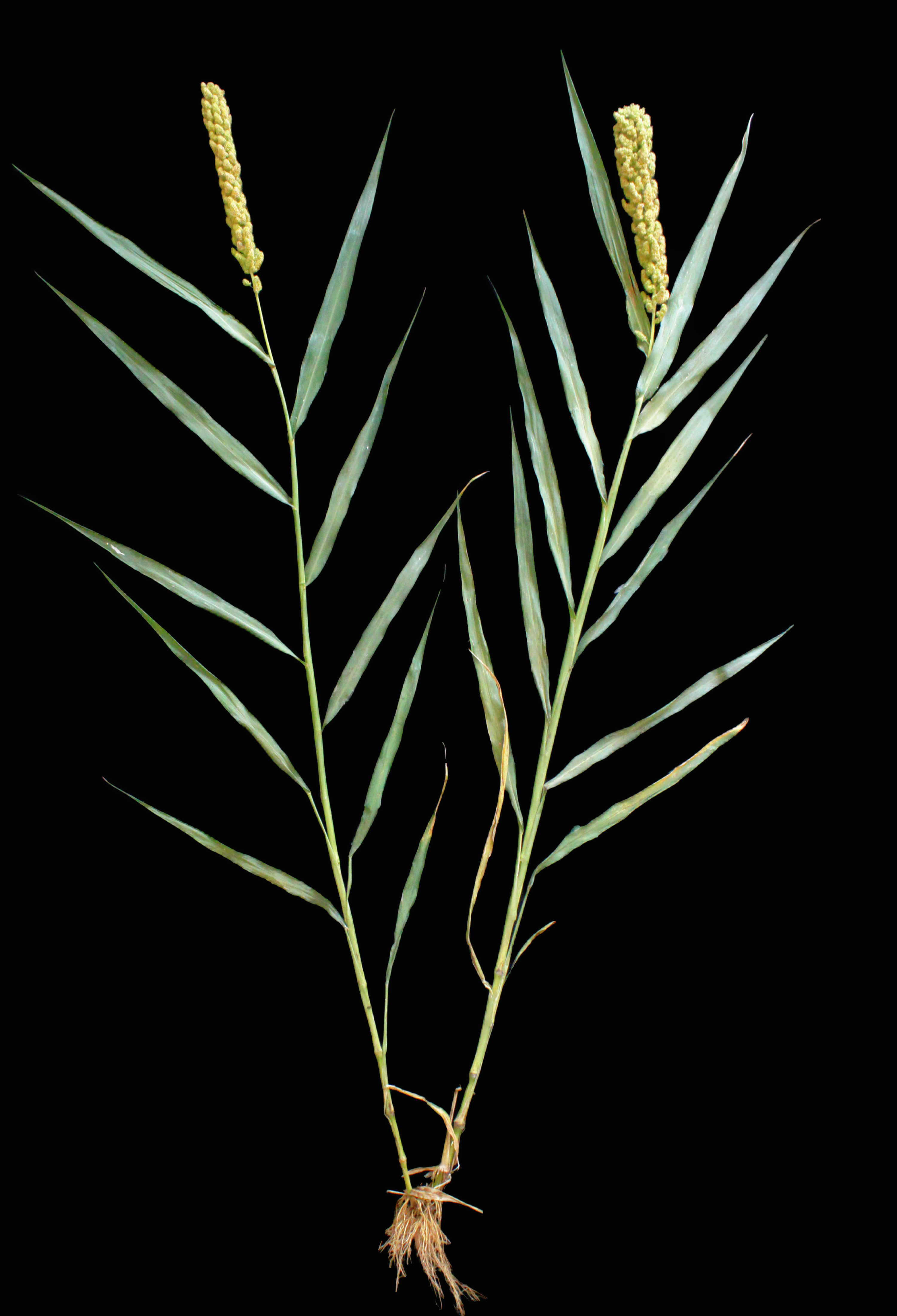


B

Fanshi, Xinzhou

113°27' E, 39°19' N


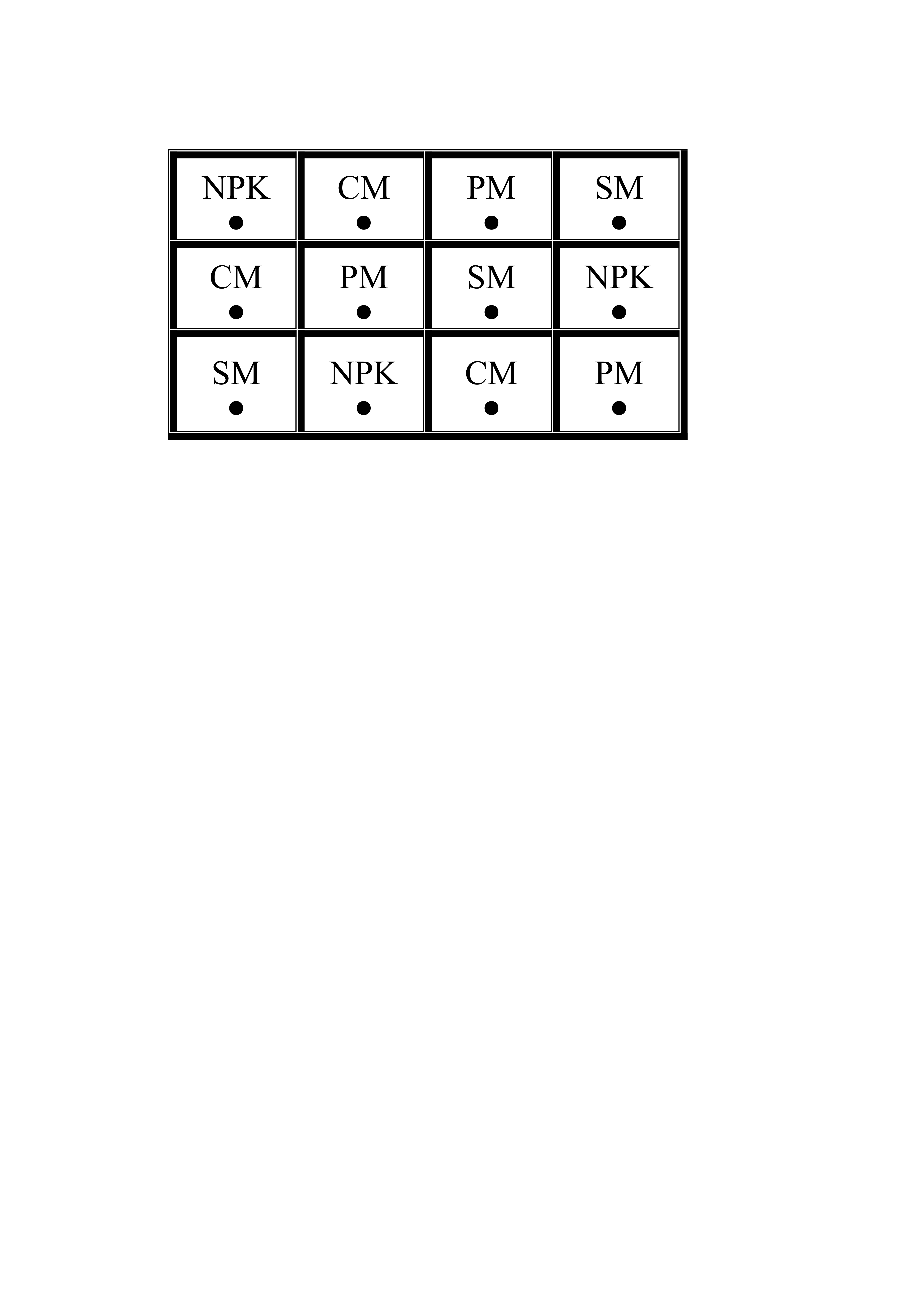


Fig. S1. Sample collection location (A) and experimental setting (B). Solid dots represent the sampling points. In each plot, soil samples were collected from five points along an "S"- shaped pattern and then mixed into a single composite sample. NPK: pure chemical fertilizer; CM: cow manure replacement; PM: pig manure replacement; SM: sheep manure replacement.


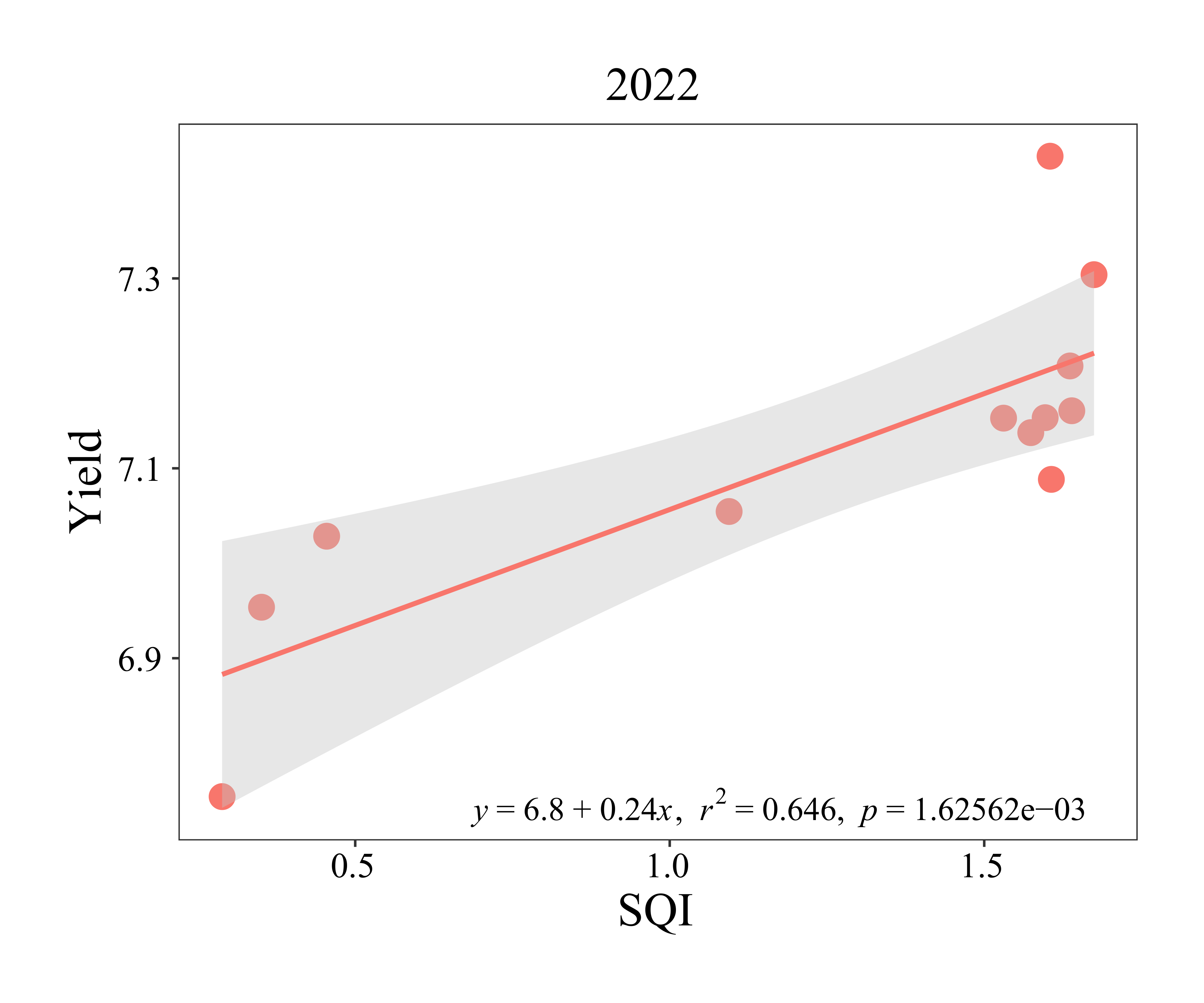

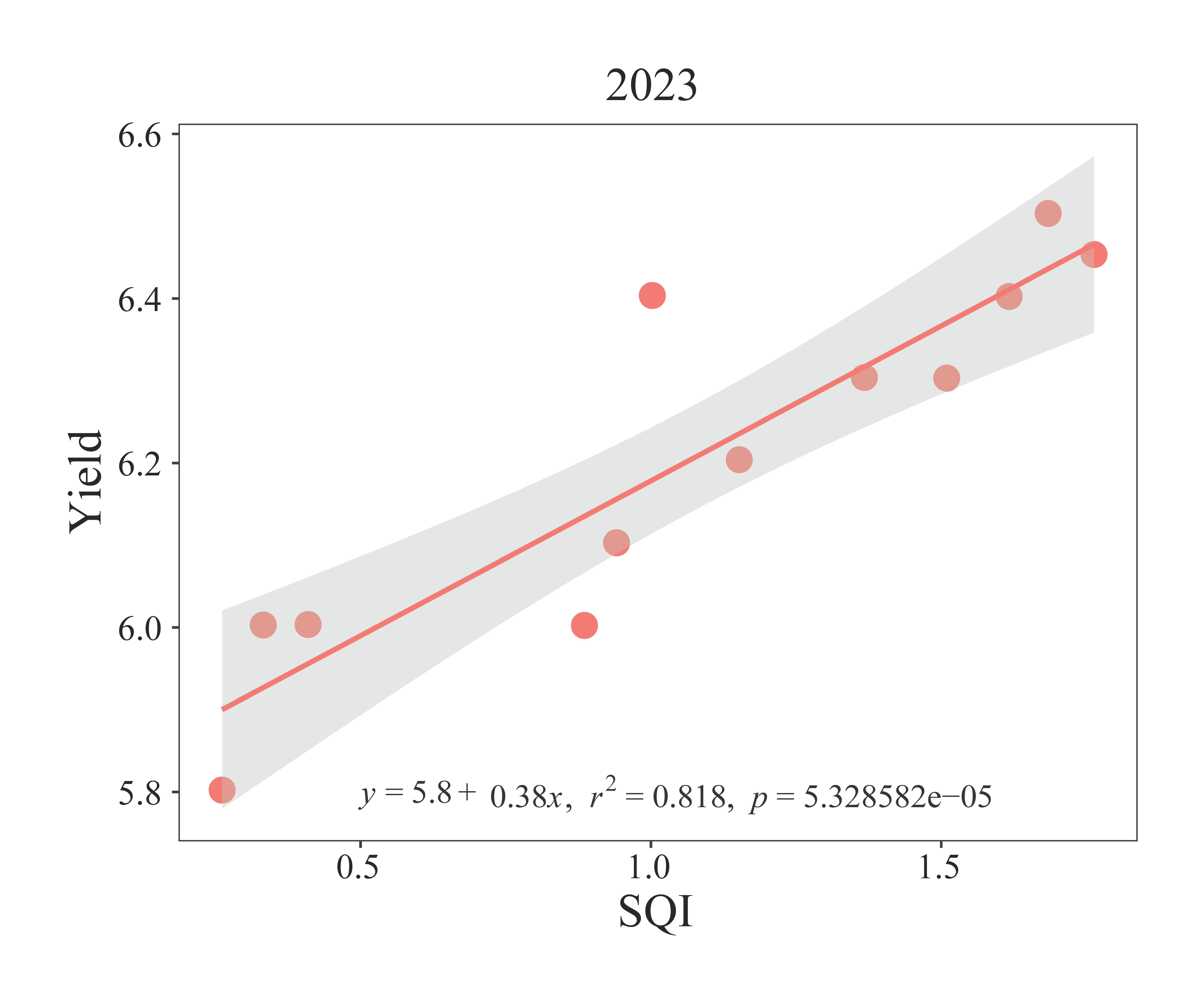

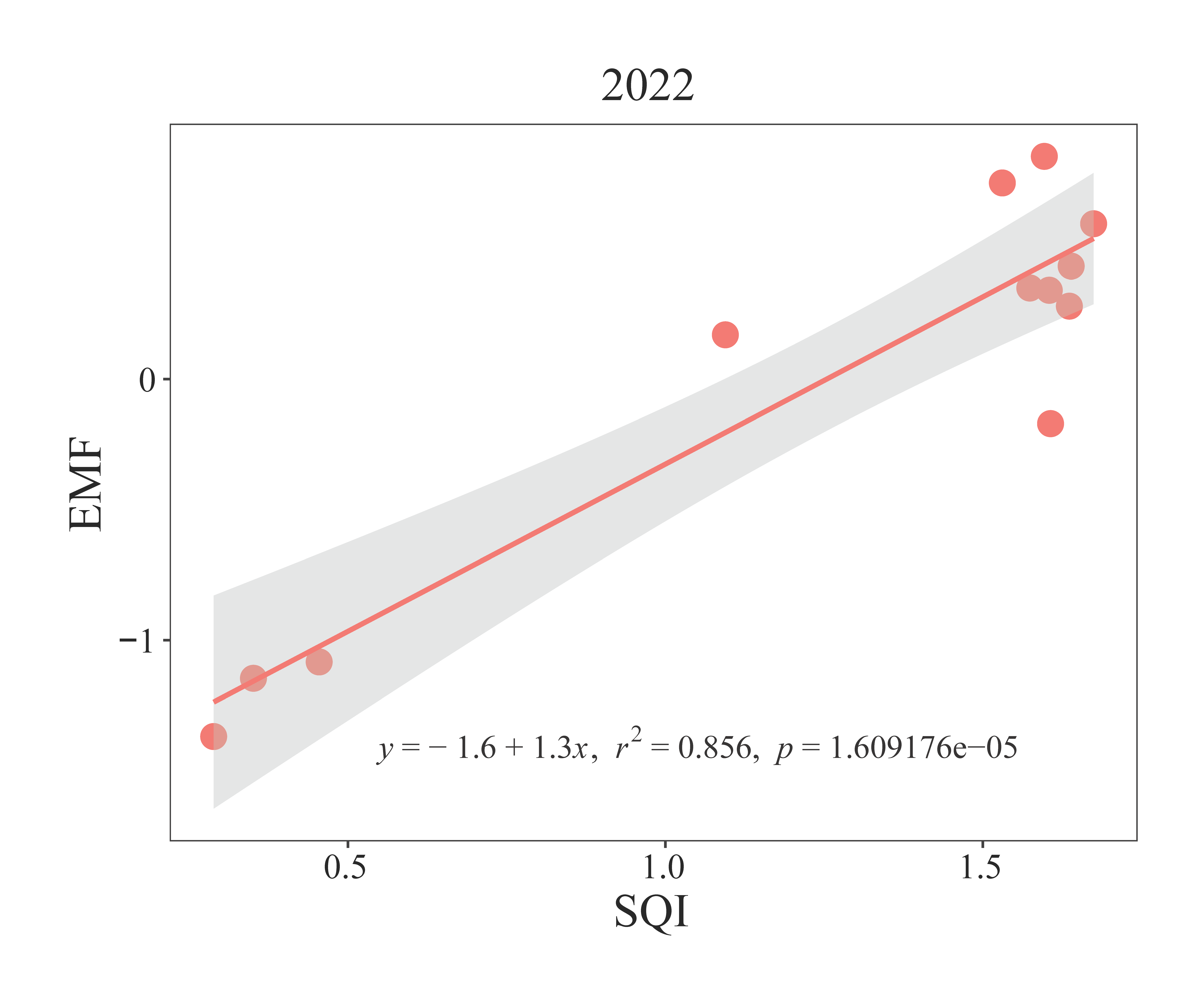


C


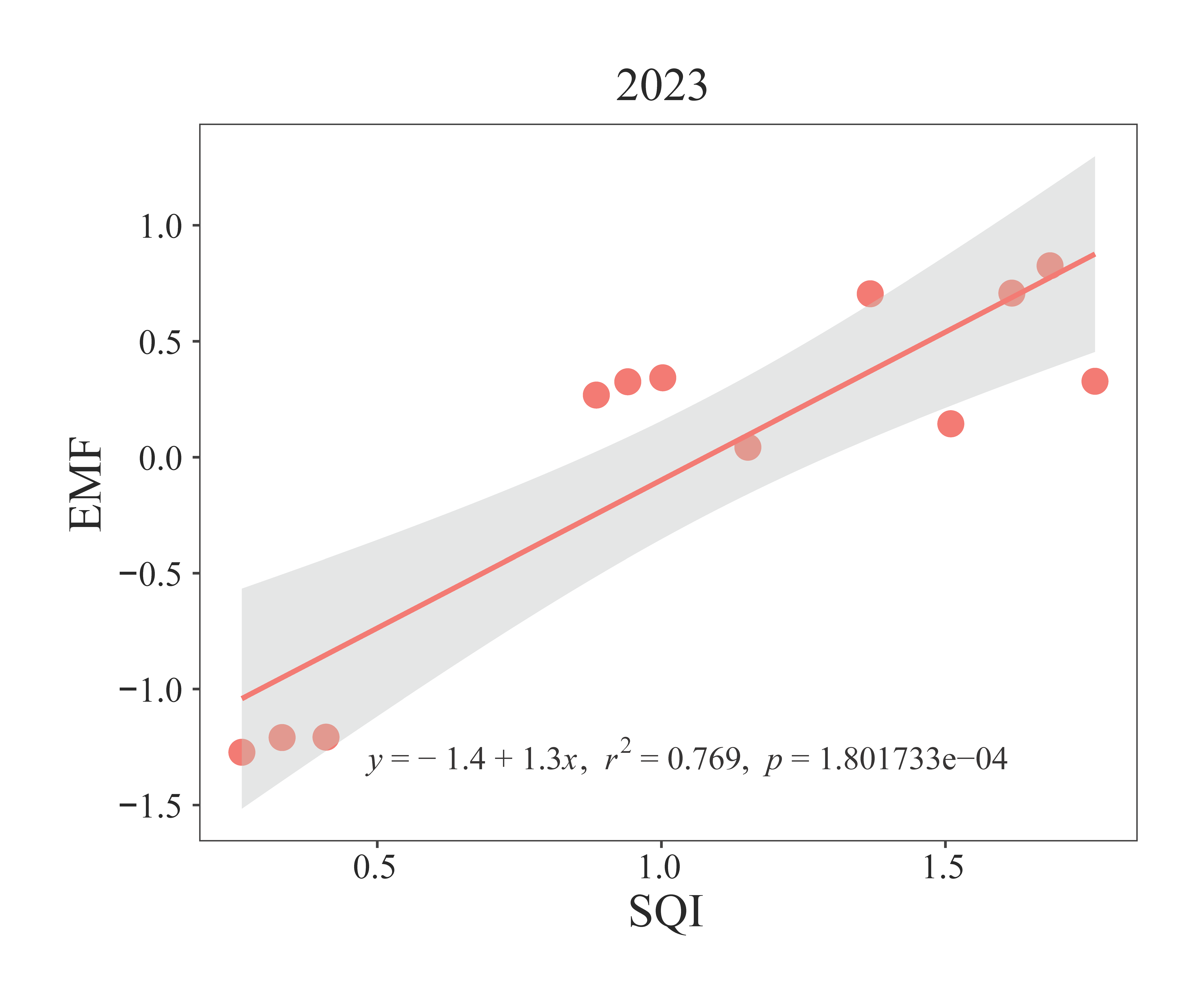


D

A

B


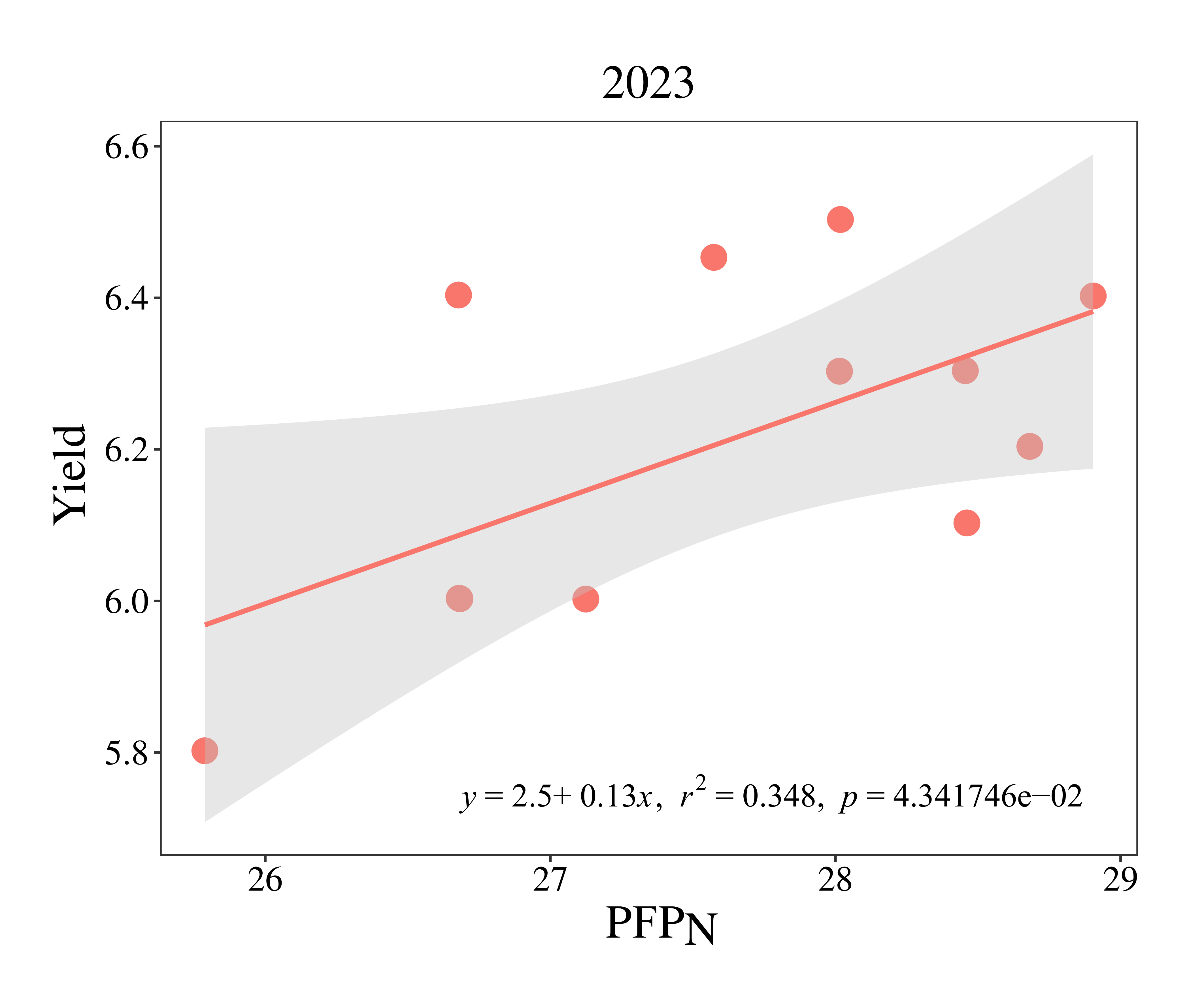


F


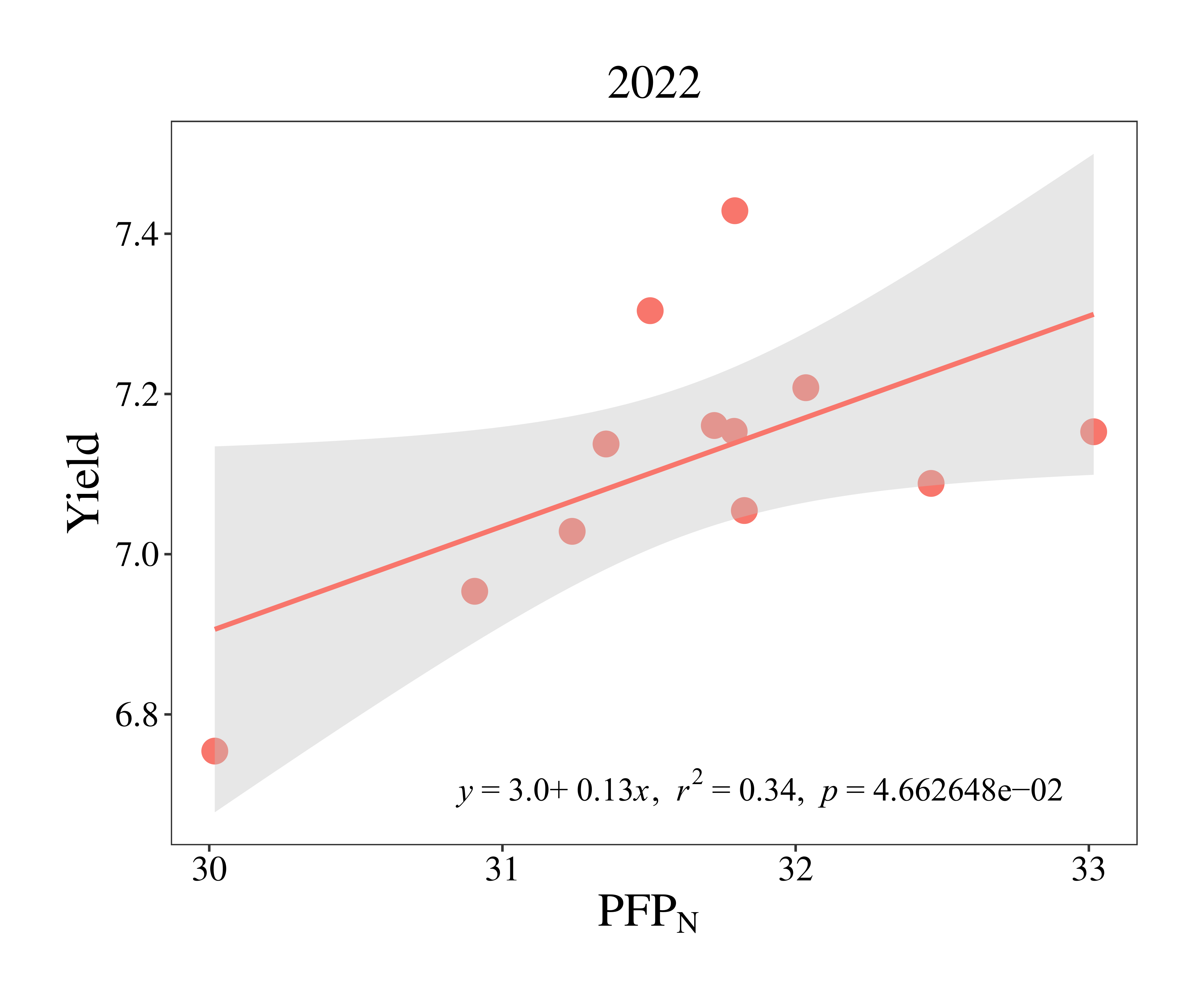


E


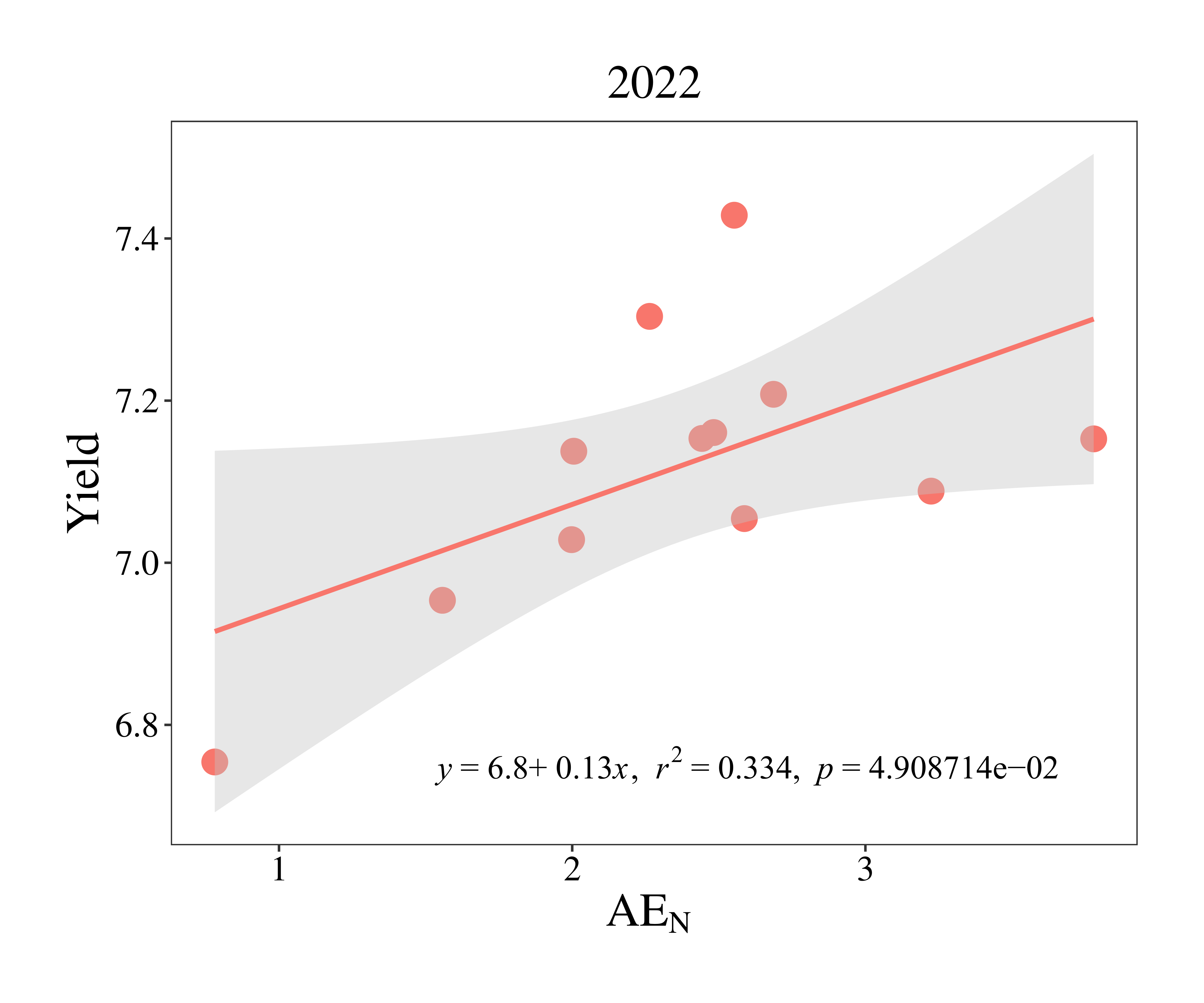


H


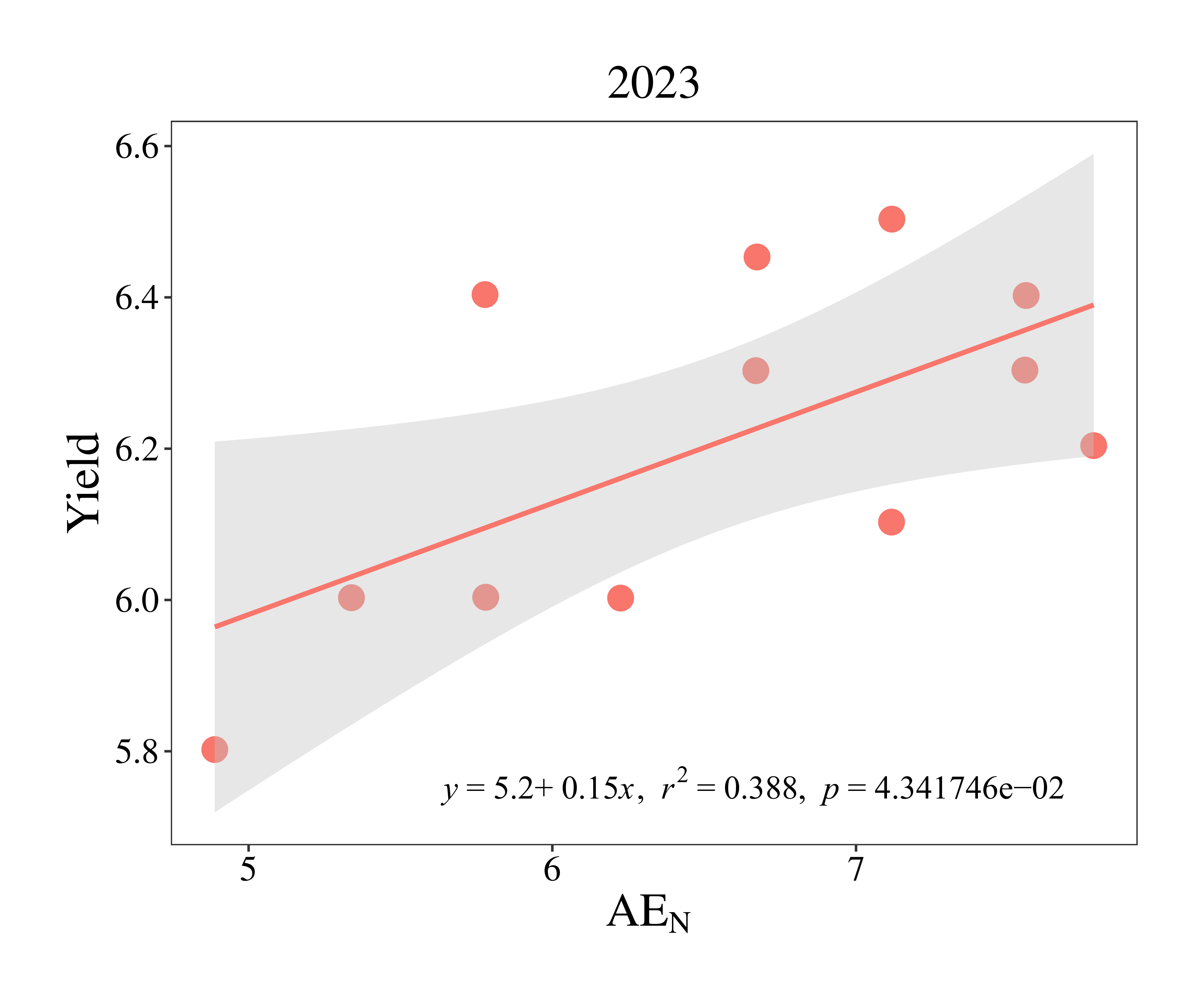


G

Fig. S2. Relationships between the soil quality index (SQI) and ecological multifunctionality (EMF; A and B) and yield (C and D), as well as the relationships between yield and nitrogen partial productivity (PFP_N_; E and F) and nitrogen agronomic efficiency (AE_N_; G and H) from 2022 to 2023. The orange lines represent the overall regression, and the shaded areas show the 95% confidence intervals of the fit.

A

2022

B

2023


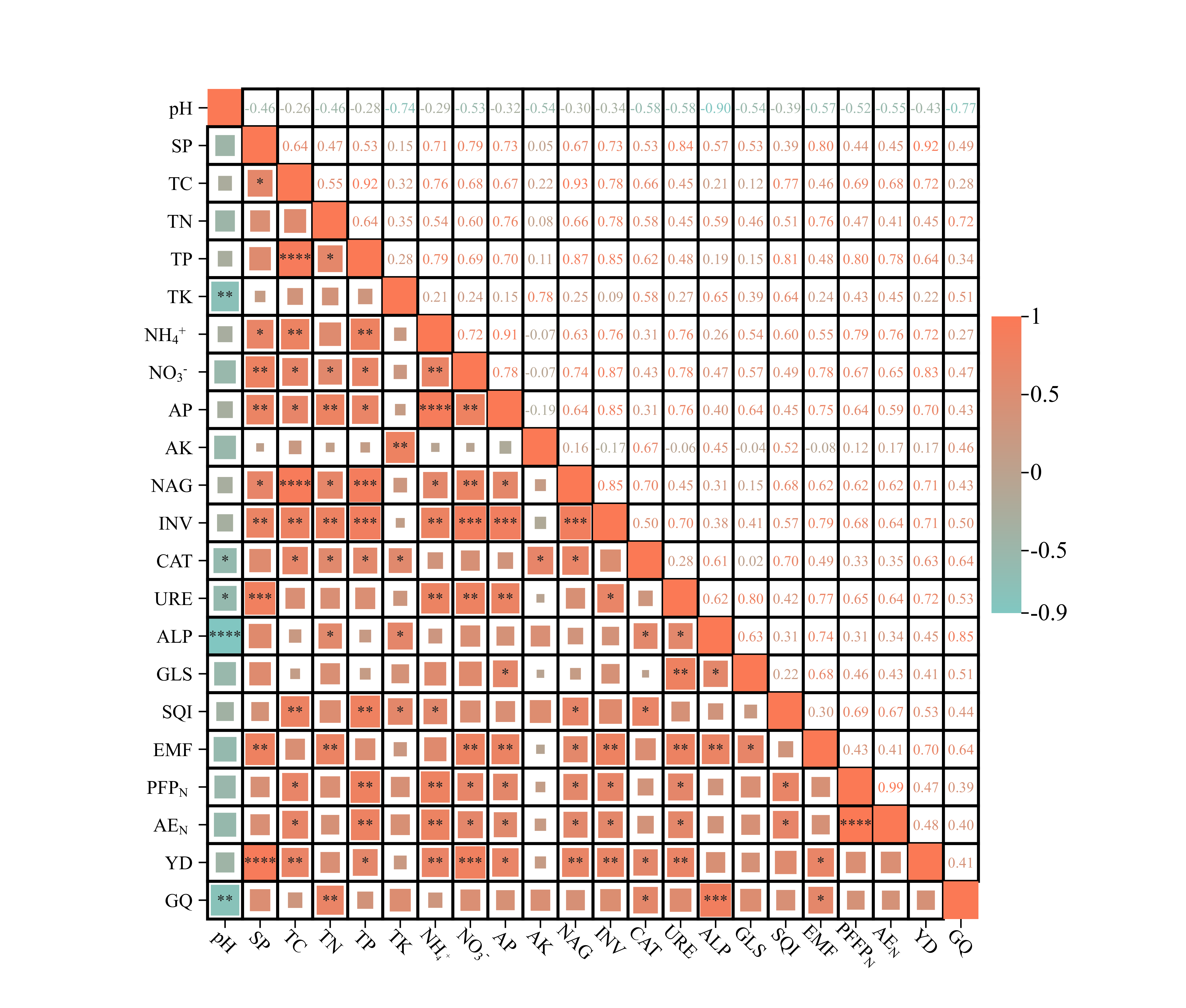

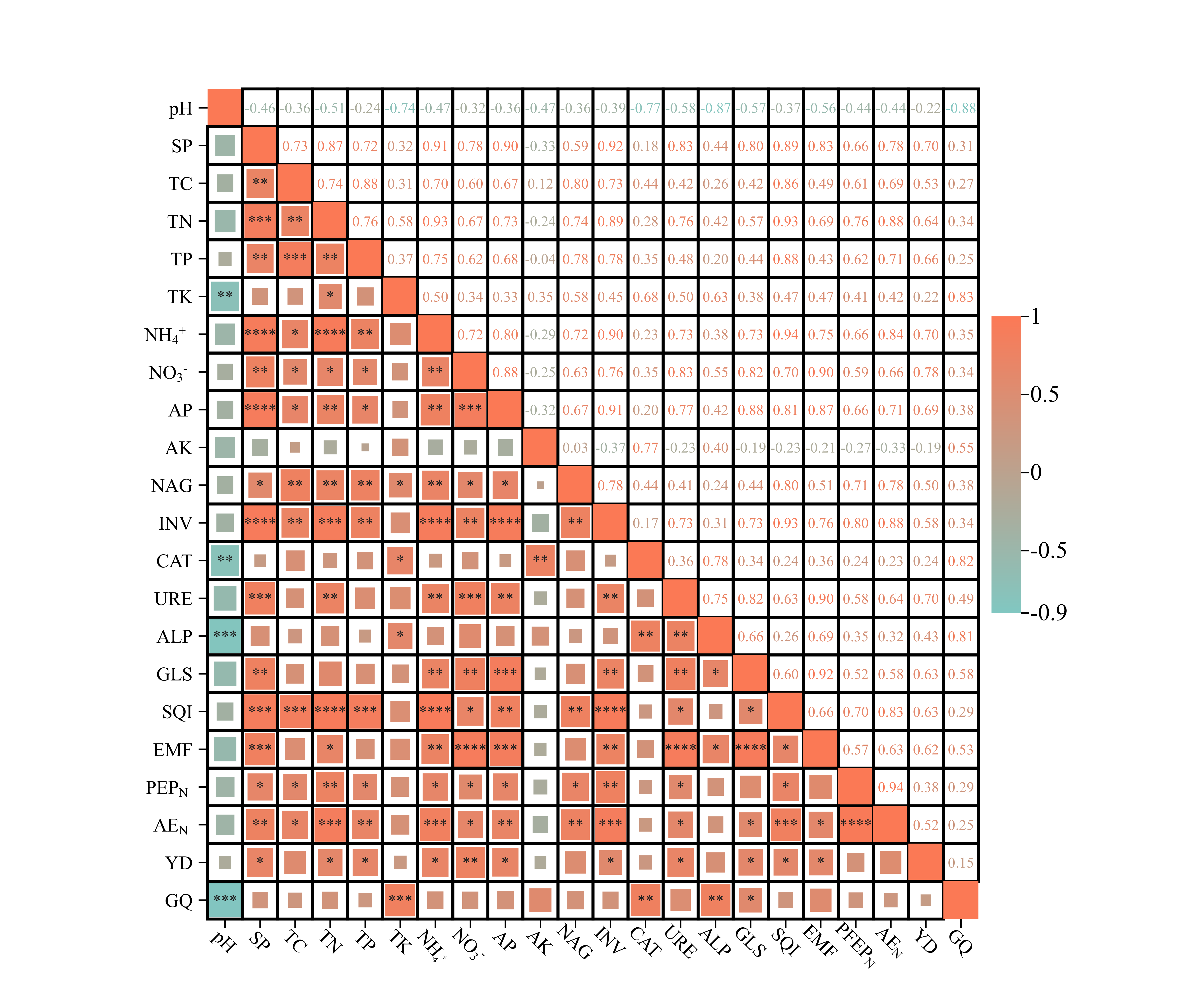


Fig. S3. The relationships among soil physicochemical properties (TC, total organic carbon; TN, total nitrogen; TP, total phosphorus; TK, total potassium; NH_4_^+^, ammonium nitrogen; NO_3_^-^, nitrate nitrogen; AP, available phosphorus; AK, available potassium), enzyme activities (ALP, soil alkaline phosphatase; NAG, β-N-acetylglucosaminidase; CAT, catalase; INV, invertase; URE, urease; GLS, glutaminase), soil quality index (SQI), ecological multifunctionality (EMF), nitrogen use efficiency (PFP_N_, N partial factor productivity; AE_N_, N agronomic efficiency), yield (YD), and grain quality (GQ, total flavonoid content) from 2022 (A) to 2023 (B). * denotes *p* < 0.05, ** denotes *p* < 0.01, *** denotes *p* < 0.001, and **** denotes *p* < 0.0001.




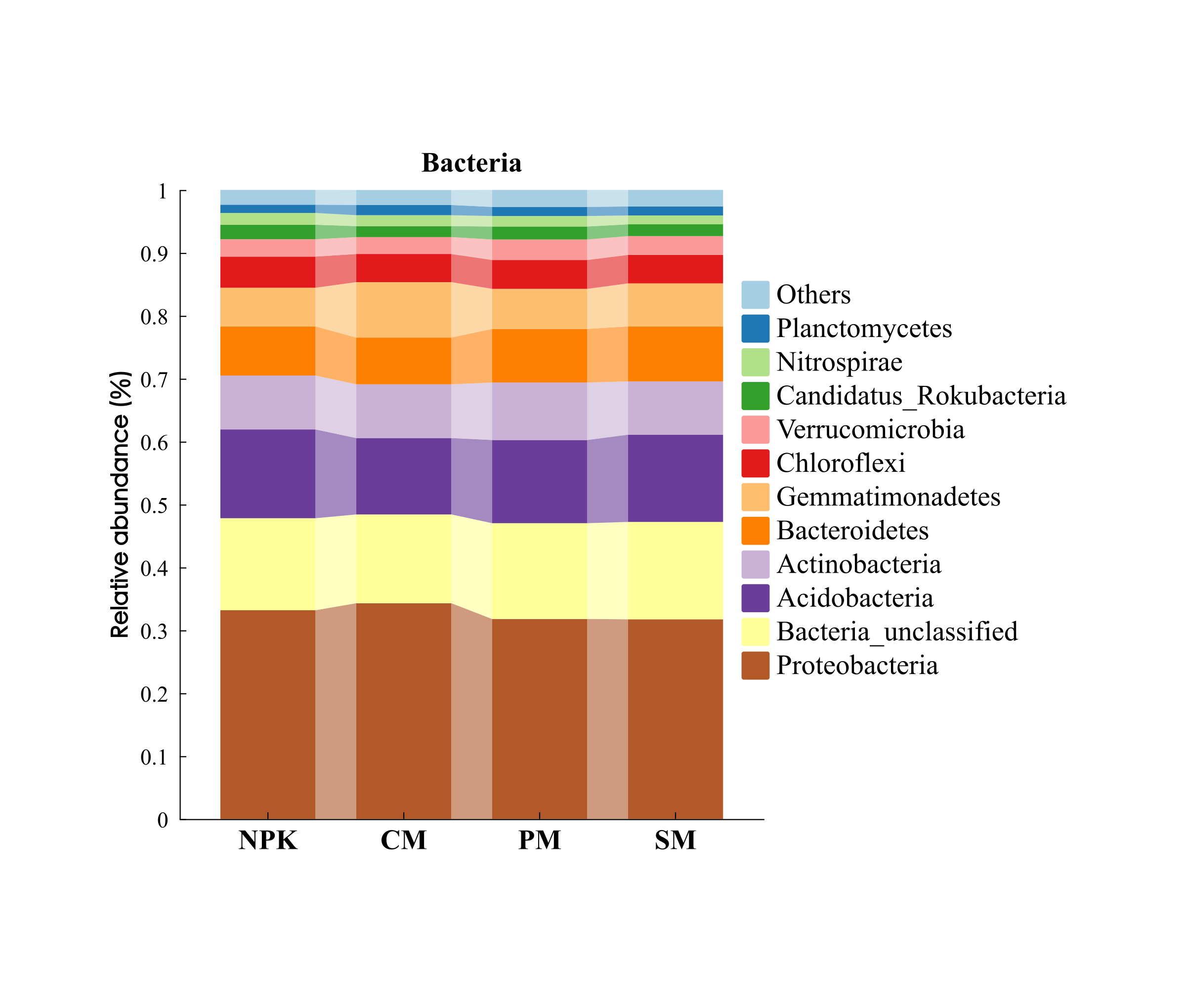


B


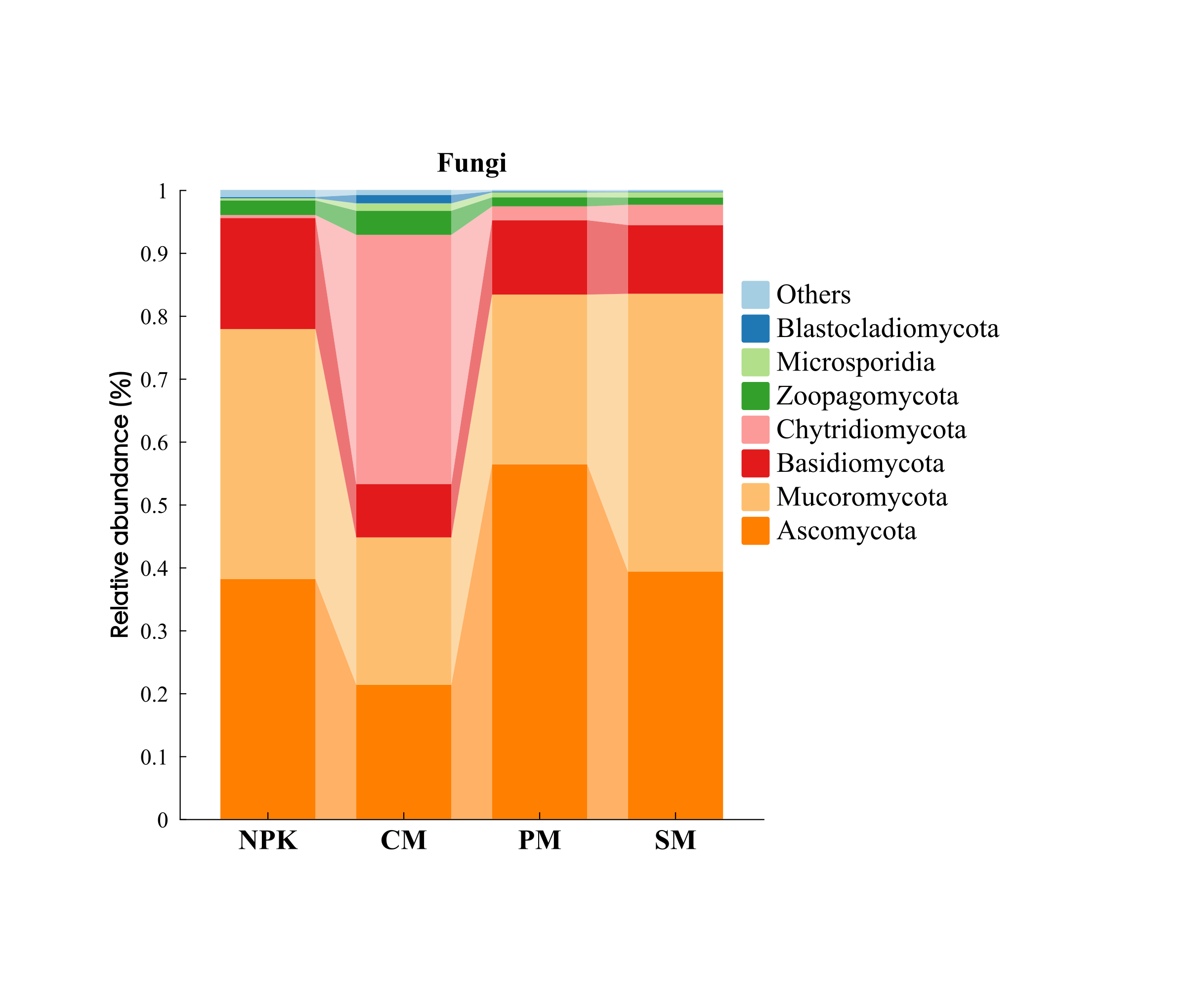


C


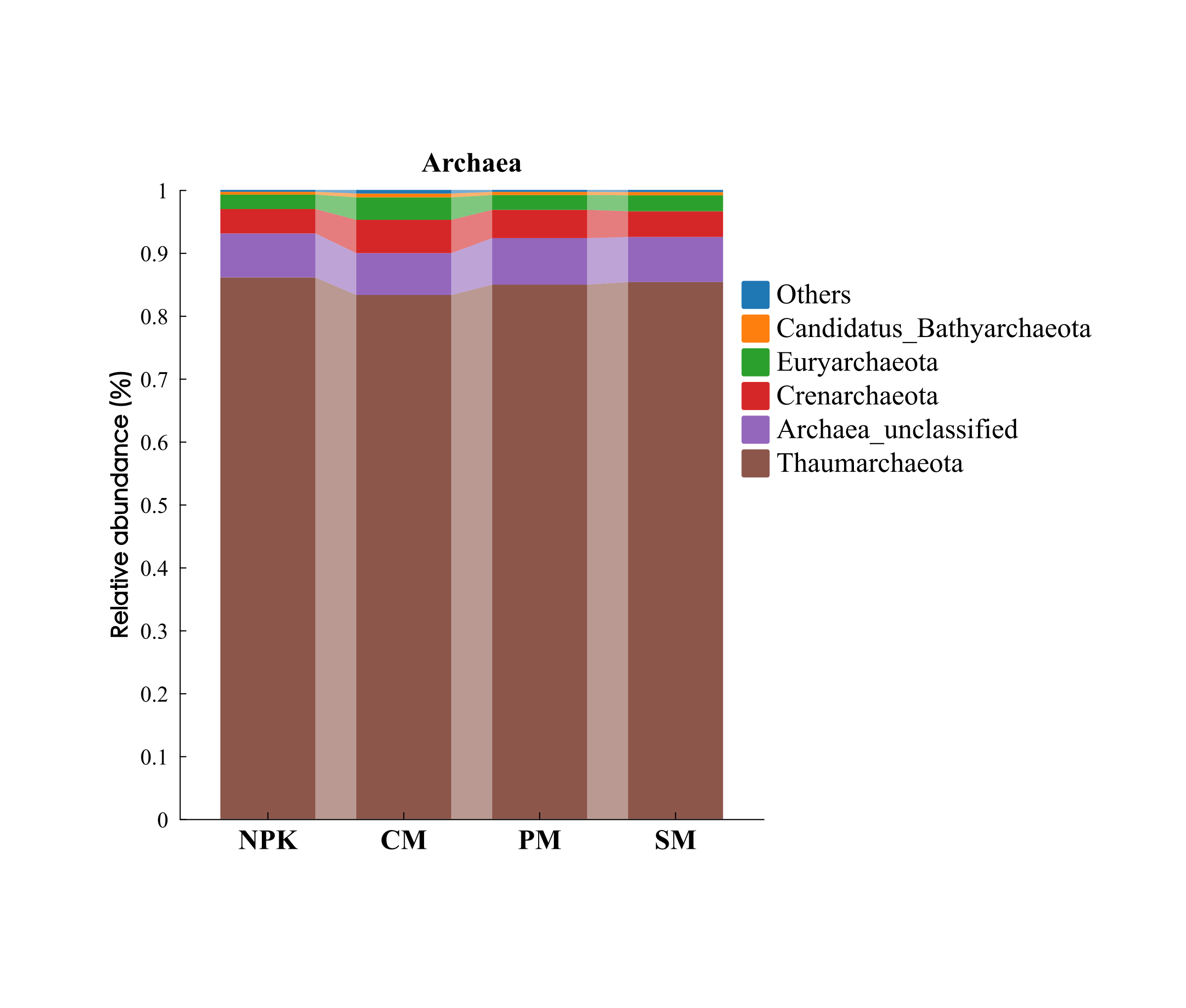


D

A

Fig. S4. Venn diagram based at the unigene level (A). The composition of bacterial (B), fungal (C), and archaeal (D) communities at the phylum level under different fertilization regimes. NPK: pure chemical fertilizer; CM: cow manure replacement; PM: pig manure replacement; SM: sheep manure replacement.


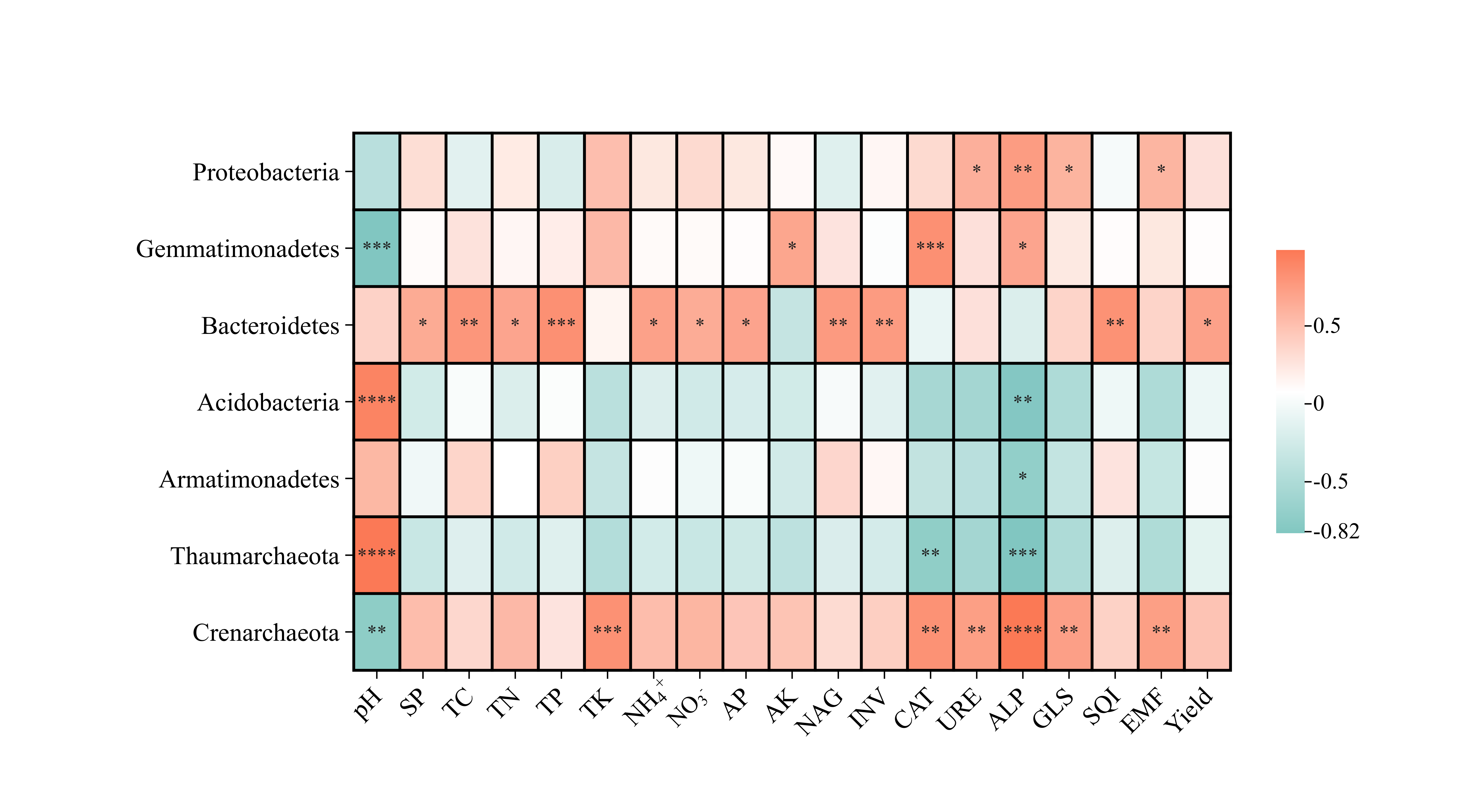


Fig. S5. Spearman's correlation analysis heat maps of soil physicochemical properties and key phyla relative abundance corresponding to the key nodes. TC, total organic carbon; TN, total nitrogen; TP, total phosphorus; TK, total potassium; NH_4_^+^, ammonium nitrogen; NO_3_^-^, nitrate nitrogen; AP, available phosphorus; AK, available potassium; ALP, soil alkaline phosphatase; NAG, β-N-acetylglucosaminidase; CAT, catalase; INV, invertase; URE, urease; GLS, glutaminase; SQI, soil quality index; EMF, ecological multifunctionality; Yield, the annual yield of foxtail millet grain in 2023. Asterisks indicate significantly different values: * *p* < 0.05; ** *p* < 0.01; *** *p* < 0.001; **** *p* < 0.0001.


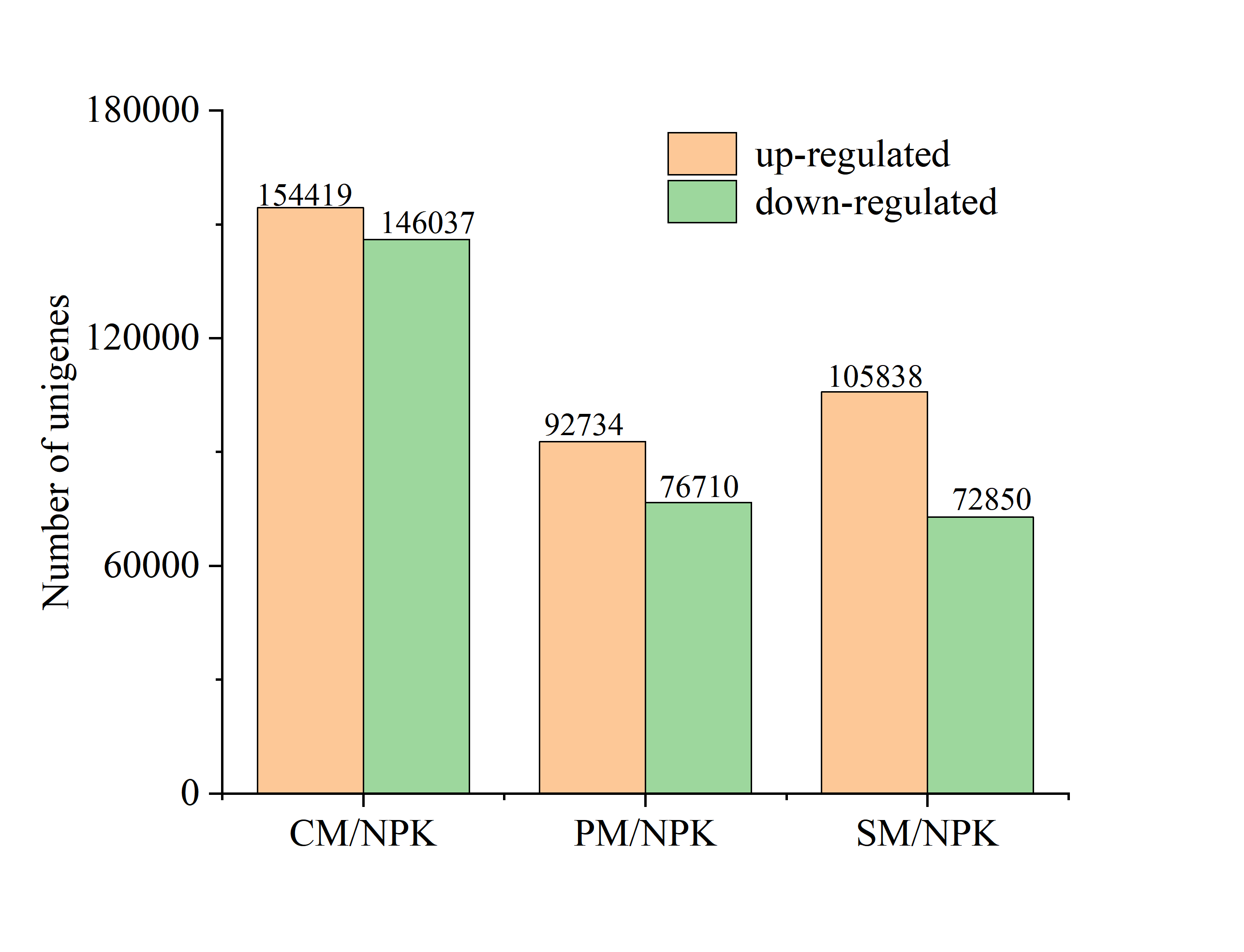


Fig. S6. The number of differentially expressed unigenes (DEGs) between the inorganic fertilizer treatment and organic replacement treatments. NPK: pure chemical fertilizer; CM: cow manure replacement; PM: pig manure replacement; SM: sheep manure replacement.


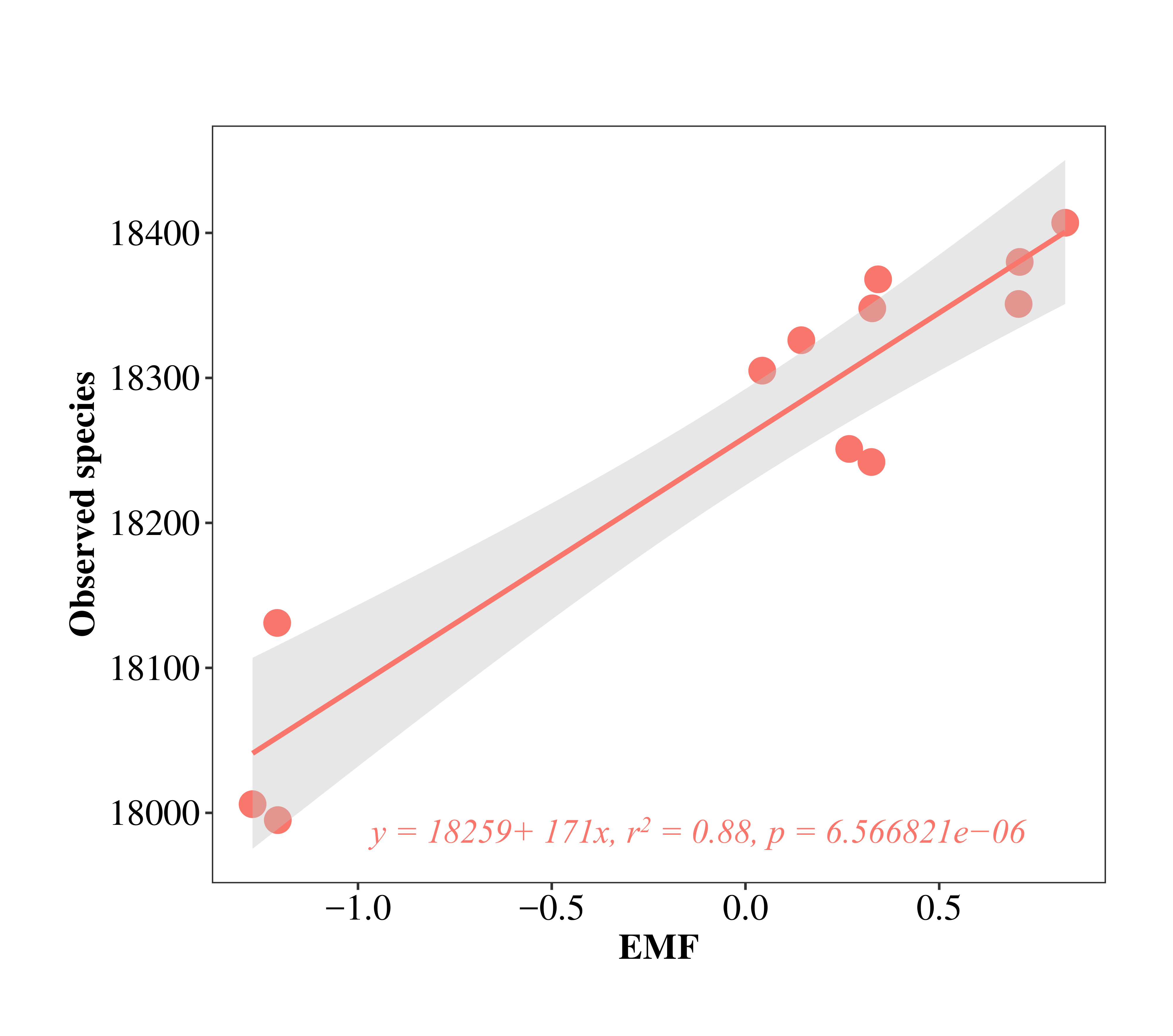

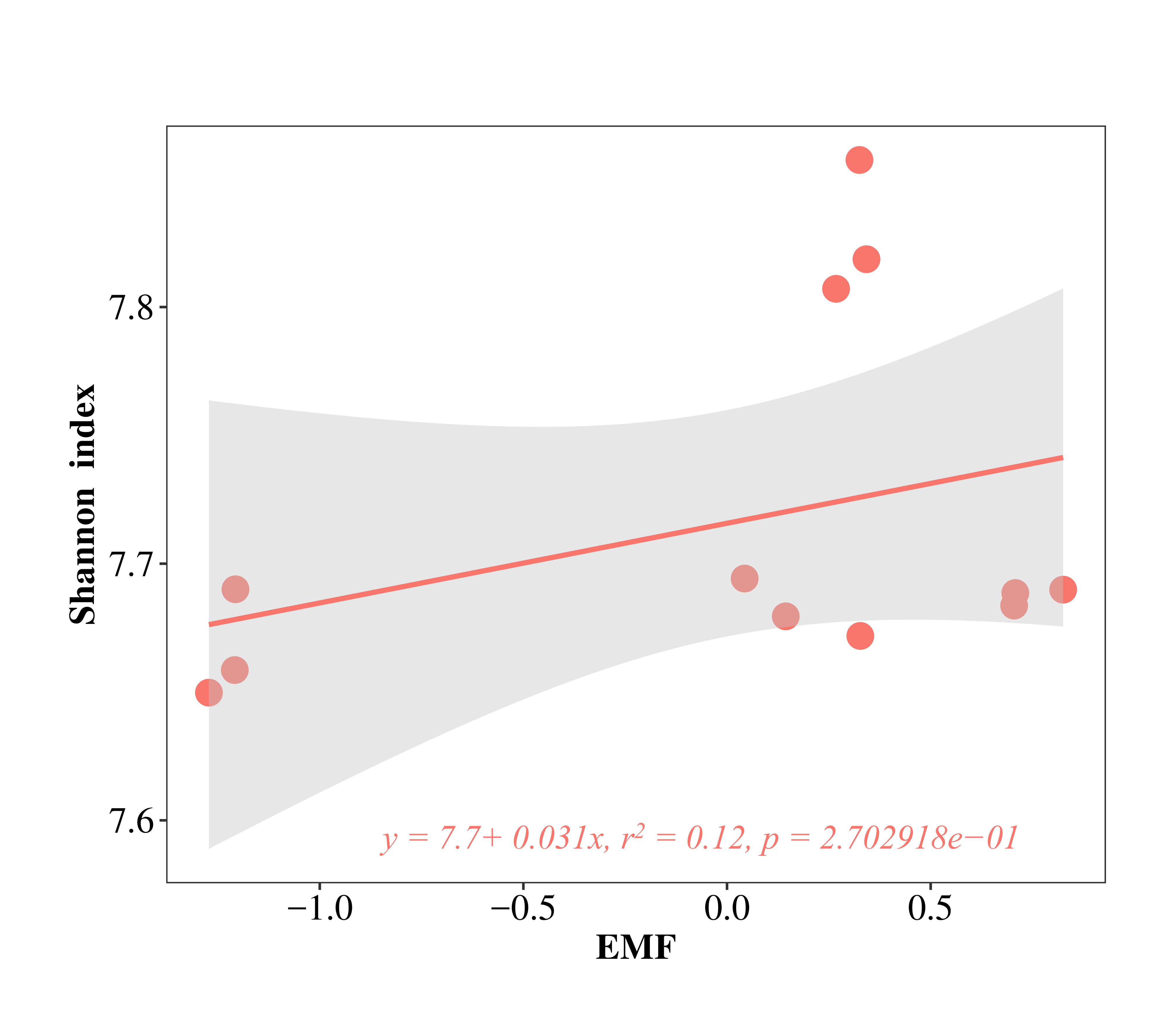

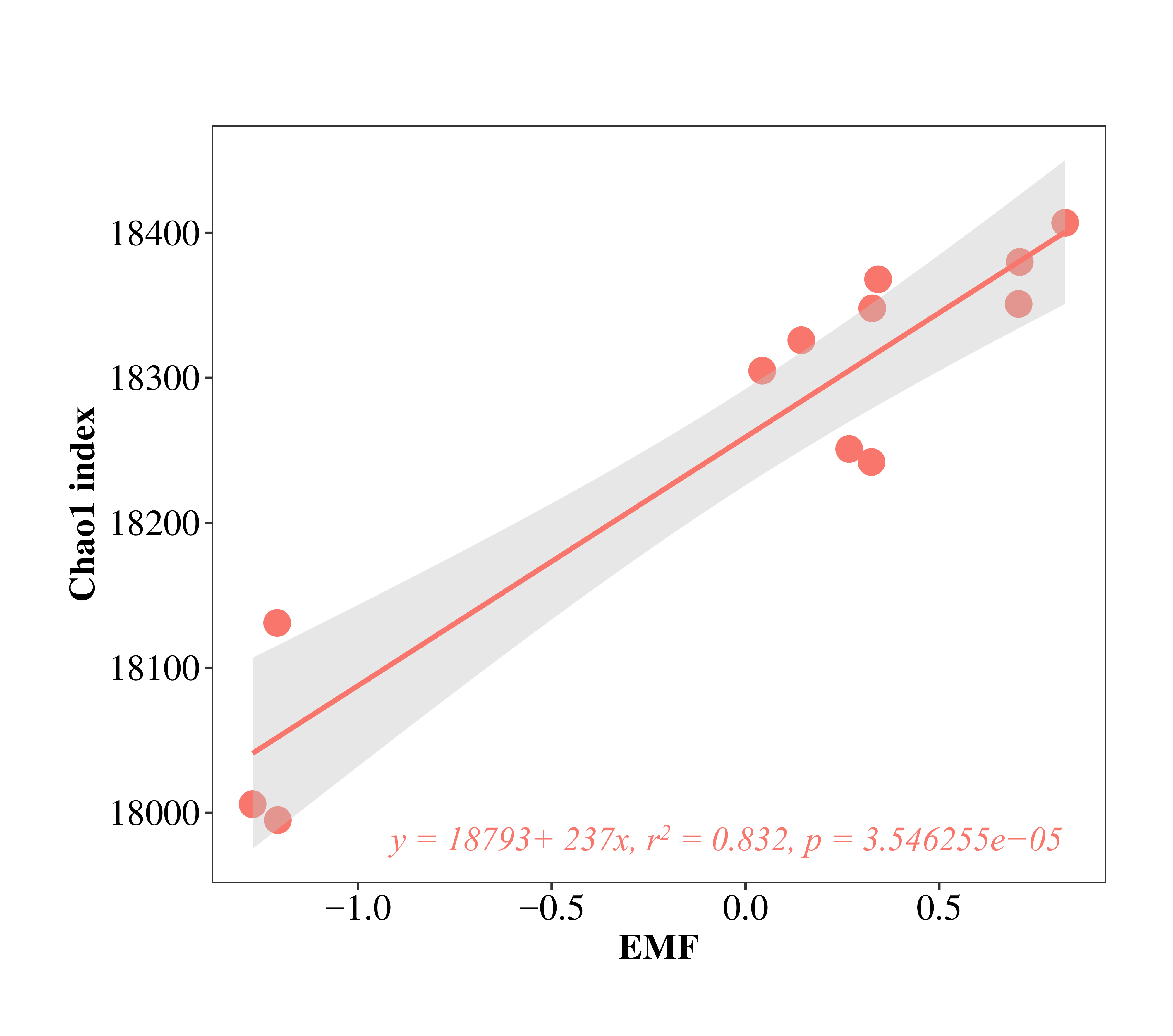


Fig. S7. The relationships between soil microbial functional diversity and EMF.
